# Photosystem II: commonality and diversity with emphasis on the extrinsic subunits

**DOI:** 10.1093/pcp/pcaf072

**Published:** 2025-07-02

**Authors:** Ko Imaizumi, Kentaro Ifuku

**Affiliations:** Division of Applied Life Sciences, Graduate School of Agriculture, Kyoto University, Kitashirakawa Oiwake-cho, Sakyo-ku, Kyoto 606-8502, Japan; Division of Applied Life Sciences, Graduate School of Agriculture, Kyoto University, Kitashirakawa Oiwake-cho, Sakyo-ku, Kyoto 606-8502, Japan

**Keywords:** extrinsic subunits, light harvesting, photosystem II, water oxidation

## Abstract

Photosystem II (PSII) is a multi-subunit complex embedded in the thylakoid membranes of all oxygenic photosynthetic organisms, ranging from cyanobacteria to algae and plants. PSII converts solar energy to chemical energy and produces oxygen by oxidizing water, thereby sustaining life on Earth. The basic structures of the PSII core and the fundamental mechanisms of light-driven water oxidation are well-conserved among the diverse oxyphototrophs. Meanwhile, the compositions of the extrinsic subunits, which have critical roles in supporting water oxidation, have largely changed during evolution. The light-harvesting antenna systems of PSII are even more diverse. In this review, we comprehensively summarize the commonality of PSII, while highlighting the diversity of PSII among various oxyphototrophs. This includes summaries on the overall PSII core structure, PSII assembly and repair, charge separation and electron transfer in PSII, water oxidation by PSII, peripheral light-harvesting antennas of PSII, and PSII–antenna supercomplex structures, as well as a summary on the extrinsic subunits. Special emphasis is given to the extrinsic subunits, updating our understanding of their roles, and discussing the structural and functional complementation of the different sets of extrinsic subunits in cyanobacterial, red-lineage, and green plant PSII.

## Introduction

Oxygenic photosynthesis is one of the most important biological processes that sustains almost all life on Earth. In this process, light energy is used to convert water and carbon dioxide into molecular oxygen and carbohydrates. The process can be divided into two parts: photosynthetic electron transport and carbon fixation. In photosynthetic electron transport, solar energy is converted to chemical energy by the light-driven oxidation of water to molecular oxygen and the reduction of NADP^+^ to NADPH, while producing ATP. The produced ATP and NADPH are then used for the fixation of carbon dioxide.

Oxygen evolution in photosynthesis occurs through light-driven water oxidation, catalyzed by photosystem II (PSII), a multi-subunit protein–pigment complex embedded in the thylakoid membranes of all oxygenic photosynthetic organisms: plants, algae, and cyanobacteria. Using light energy, PSII oxidizes water to molecular oxygen and reduces plastoquinone (PQ) to plastoquinol. Therefore, this complex is also known as the light-driven water:plastoquinone oxidoreductase (Wydrzynski and Satoh 2005). The electrons, extracted from water and passed to PQ, are sequentially transferred from plastoquinol to cytochrome (Cyt) *b*_6_*f*, plastocyanin (or Cyt *c*_6_), and photosystem I (PSI). PSI uses light energy to transfer these electrons to ferredoxin (Fd), which can be used to reduce NADP^+^ to NADPH via ferredoxin-NADP^+^ reductase (FNR). The names, photosystem I and II, derive from the crucial experiment by Duysens et al. (1961) which proved that two photosystems work in series, in line with predictions by Rabinowitch (1945). Illumination of ‘light 1’ with a longer wavelength, exciting a photochemical pigment system (system 1, now known as PSI), led to oxidation of Cyt *f*, and when ‘light 2’ with a shorter wavelength, proposed to excite a different photochemical pigment system (system 2, now known as PSII), was added, reduction of Cyt *f* was observed (Duysens et al. 1961). The history of research on PSII has been excellently summarized (Govindjee et al. 2017).

PSII has enabled the abundantly available water to serve as the primary electron source for photosynthetic electron transfer, and has produced most of the oxygen in the atmosphere. Oxygenic photosynthesis is estimated to have evolved at least 2.5 to 3.5 billion years ago, and the emergence of PSII had significant impacts on the environment of the Earth. The increase of atmospheric oxygen concentration, especially pronounced during the Great Oxidation Event (GOE), has enabled the development of organisms using aerobic respiration. The increased atmospheric oxygen also led to the formation of the ozone layer, which shields the Earth’s surface from UV radiation, enabling the development of life on land. The vital contribution of PSII to the development and sustenance of diverse life forms on Earth is emphasized by the reference to this complex as the ‘engine of life’ (Barber 2003). The impacts of PSII and oxygenic photosynthesis on life on Earth have been well described in (Shevela et al. 2023) and references therein.

Today, diverse oxygenic photosynthetic organisms (oxyphototrophs) are found ubiquitously across various habitats on Earth. Primary endosymbiosis, wherein a cyanobacterium was engulfed by a eukaryote, gave rise to Archaeplastida (primary plastid-containing eukaryotes), formed by Rhodophyta (red algae), Viridiplantae (green plants: green algae and land plants), and Glaucophyta (Keeling 2010). Additional rounds of endosymbiotic events have led to further diversification of oxyphototrophs. PSII has mostly been studied in cyanobacteria, red-lineage oxyphototrophs (red algae and red-lineage algae [algae possessing red algal-derived plastids]), and green plants, and partially in glaucophytes. The basic structures of the PSII core, as well as the fundamental mechanisms of water splitting by PSII, are conserved among all oxyphototrophs. However, some aspects of PSII are diverse, such as the extrinsic subunits and peripheral light-harvesting antenna systems. In this review, we broadly summarize the basics of the structures and functions of PSII, with attention to the commonality and diversity of PSII among various oxyphototrophs. Particular emphasis will be placed on the extrinsic subunits, with an effort to substantially update our previous understanding.

## Overview of the Structure and Assembly of PSII

### Overall structure of PSII

**Fig. 1:**
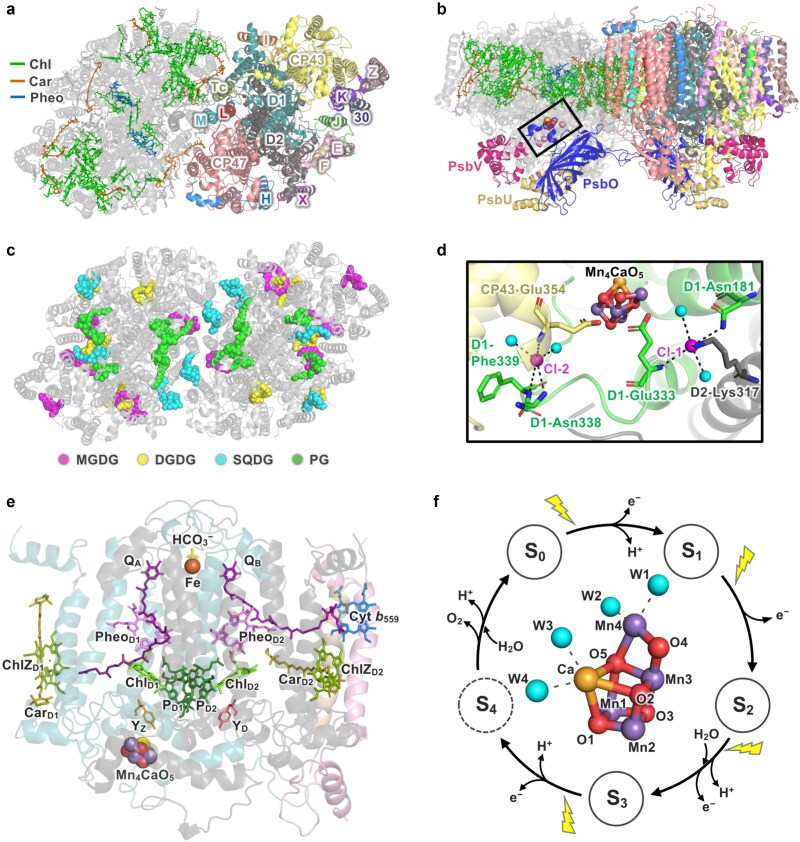
Overview of the PSII core. (a–c) Subunit composition of cyanobacterial PSII and the locations of pigments, lipids, and the Mn_4_CaO_5_ cluster in PSII (PDB ID: 3WU2). (a) Locations of the membrane-intrinsic subunits are shown in one monomer, and the locations of pigments are shown in the other monomer, viewed from the stromal/cytosolic side of PSII. Subunits are labeled according to the protein nomenclature (e.g. E for PsbE). Note that D1, CP47, CP43, and D2 are encoded by the *psbA*, *psbB*, *psbC*, and *psbD* genes, respectively. Extrinsic subunits were removed for clarity. (b) Side view of PSII in panel a, with the membrane-extrinsic subunits (PsbO, PsbV, and PsbU) shown. In one monomer, the Mn_4_CaO_5_ cluster and Cl^−^ ions are shown, with this region indicated by a black box. (c) Locations of lipids viewed from the stromal/cytosolic side of PSII. (d) Locations of the two Cl^−^ ion binding sites near the Mn_4_CaO_5_ cluster (PDB ID: 4UB6). D1, D2, and CP43 are shown in green, gray, and yellow cartoon views, respectively. Cl^−^ ions are shown as pink spheres, water molecules near the Cl^−^ ions are shown as blue spheres, and the amino acid residues associated with the Cl^−^ ions are shown as stick models. (e) Electron transfer cofactors, other commonly found redox-active cofactors, and related components in the reaction center of a PSII monomer are shown (PDB ID: 3WU2). D1, D2, PsbE, and PsbF subunits are shown as blue, gray, pink, and beige cartoon views, respectively. P680 (reaction center chlorophylls P_D1_, P_D2_, Chl_D1_, and Chl_D2_), pheophytins (Pheo_D1_ and Pheo_D2_), PQs (Q_A_ and Q_B_), redox active tyrosines (Y_Z_ and Y_D_), extra chlorophylls (ChlZ_D1_ and ChlZ_D2_), associated carotenoids (Car_D1_ and Car_D2_), the bicarbonate ion, and the heme of Cyt *b*_559_ are shown as stick models. The Mn_4_CaO_5_ cluster and the non-heme iron are shown as spheres. (f) The S-state cycle for water oxidation and an enlarged view of the Mn_4_CaO_5_ cluster (PDB ID: 4UB6). Mn, Ca, and O atoms are shown as purple, orange, and red spheres, respectively. The blue spheres indicate water molecules; W1 and W2 are water molecule ligands to Mn4, and W3 and W4 are those to Ca.

PSII is a large complex, generally present as a dimer when in an active form. Each PSII monomer consists of more than 20 subunits (Shen et al. 2021): 17 to 19 membrane intrinsic subunits and 3 to 5 membrane extrinsic subunits that bind on the lumenal side of PSII (Fig. 1a and 1b). It contains many pigments (chlorophylls [Chl], carotenoids [Car], and pheophytins [Pheo]) and lipids, as well as PQ, heme and non-heme iron, the Mn_4_CaO_5_ cluster, and bicarbonate and chloride (Cl^−^) ions (Umena et al. 2011, Shen 2015) (Fig. 1). In addition, PSII binds peripheral antenna proteins that enable efficient light-harvesting as well as dissipation of excess energy (Larkum 2020).

Two transmembrane subunits, D1 and D2, forming a heterodimer, constitute the reaction center core of PSII. All cofactors directly involved in charge separation, water oxidation, and electron transfer to PQ are associated with this heterodimer (Fig. 1e). Membrane intrinsic Chl binding proteins, CP43 and CP47, bind to the two sides of the D1/D2 heterodimer and serve as inner antennas. At the same time, their lumenal domains are also important for water oxidation. Cytochrome (Cyt) *b*_559_, formed by the membrane intrinsic PsbE and PsbF subunits, is also an essential component of PSII. While it has critical structural roles in the biogenesis and stable accumulation of PSII, no consensus has been made whether it also has physiologically relevant redox functions (Shinopoulos and Brudvig 2012, Chu and Chiu 2016, Chiu and Chu 2022, Cramer and Zakharov 2022, Imaizumi et al. 2025a). The other membrane intrinsic subunits have a molecular weight of mostly less than 10 kDa and are thus often referred to as the low molecular weight (LMW) subunits, together with PsbE and PsbF. Their roles have been summarized recently (Shen et al. 2021). The structures and functions of membrane extrinsic subunits are summarized in detail in a later section.

### PSII assembly and repair

*De novo* assembly of PSII initiates with the binding of D2 to Cyt *b*_559_, forming the D2 module (D2_mod_). The D1 module (D1_mod_), consisting of D1 and PsbI, binds to the D2_mod_, resulting in the formation of the reaction center complex of PSII (Nanba and Satoh 1987, Webber et al. 1989). Subsequently, the CP47 assembly module binds to form the RC47 complex (CP43-less intermediate), and further binding of the CP43 assembly module leads to the formation of the PSII core complex. This is followed by the assembly of the Mn_4_CaO_5_ cluster, binding of membrane extrinsic subunits to the lumenal side of PSII, and dimerization of PSII. Many assembly factors are involved during the briefly described process above. Assembly of PSII has been reviewed in (Nickelsen and Rengstl 2013, Komenda et al. 2024).

PSII undergoes frequent photooxidative damage, and oxyphototrophs cope with this through the PSII repair cycle (Aro et al. 1993). The cycle initiates with the partial disassembly of the damaged PSII dimer, followed by degradation of damaged D1, insertion of newly synthesized D1, and reassembly of PSII. In plants, phosphorylation of PSII core subunits by STN8 is suggested to be involved in the facilitation and fine-tuning of PSII repair (Bonardi et al. 2005, Tikkanen and Aro 2012, Kato and Sakamoto 2014). Although the D1 subunit is most prone to photodamage, other subunits such as D2 and CP43 can also be damaged in addition to D1, especially under stress conditions (Yao et al. 2012). Therefore, while ‘fast repair’ of PSII by the replacement of D1 is most well-known, ‘slow repair’ of PSII by replacing multiple subunits also occurs (Komenda et al. 2024). When the rate of PSII damage exceeds the capacity of PSII repair, photoinhibition is observed (Aro et al. 1993, Murata et al. 2007).

### Lipids in PSII

PSII also contains many lipid molecules. Four classes of lipids, also comprising the thylakoid membranes, are specifically bound within PSII (Fig. 1c and Supplementary Fig. S1): the galactolipids monogalactosyldiacylglycerol (MGDG) and digalactosyldiacylglycerol (DGDG), the sulfolipid sulfoquinovosyldiacylglycerol (SQDG), and the phospholipid phosphatidylglycerol (PG) (Mizusawa and Wada 2012). Some, but not all, of these lipids have conserved binding sites among PSII from various oxyphototrophs (Sheng et al. 2018, Yoshihara and Kobayashi 2022). In cyanobacteria and green plants, the lipids within PSII tend to be distributed asymmetrically across the thylakoid membrane (Kern and Guskov 2011, Sheng et al. 2018, Yoshihara and Kobayashi 2022): the headgroups of anionic lipids (SQDG and PG) are mostly oriented towards the stromal/cytosolic side, while those of neutral galactolipids (MGDG and DGDG) are mostly oriented towards the lumenal side. However, such asymmetry is less apparent in red algal and red-lineage algal PSII cores or supercomplexes (Supplementary Fig. S1). The lipids in PSII have crucial roles such as mediating protein–protein interactions and monomer–monomer interactions, as has been extensively reviewed (Kern and Guskov 2011, Mizusawa and Wada 2012, Sheng et al. 2018, Yoshihara and Kobayashi 2022).

## Charge separation and electron transfer in PSII

In PSII supercomplexes, the absorption of light energy by pigments (chlorophylls or phycobilins), mostly in the peripheral light-harvesting antennas, is followed by excitation energy transfer to the reaction center of PSII. When the excitation energy reaches the primary electron donor P680, P680 transitions to its excited state (P680^*^), and charge separation occurs, leading to the formation of P680^+^ and Pheo_D1_^−^. The oxidative P680^+^ drives water oxidation at the Mn_4_CaO_5_ cluster via a redox-active tyrosine residue Y_Z_ (D1-Tyr161). Meanwhile, the electron is transferred from Pheo_D1_ to the non-exchangeable plastoquinone Q_A_, and further to the exchangeable plastoquinone Q_B_ (Fig. 1e). Upon accepting a second electron, Q_B_, doubly reduced and protonated to become plastoquinol Q_B_H_2_, dissociates from the Q_B_-site of PSII, thus entering the PQ pool in the thylakoid membrane.

Each of the processes described above has been intensively studied. P680, named for its absorption peak at approximately 680 nm (Rabinowitch and Govindjee 1965), was initially recognized as the special pair of chlorophylls P_D1_ and P_D2_, in analogy with the special pair pigments in reaction centers from purple bacteria (Cardona et al. 2012). It has been accepted that charge separation results in the formation of P_D1_^+^Pheo_D1_^−^ (or [P_D1_P_D2_]^+^ Pheo_D1_^−^). However, a number of studies have suggested that the primary electron donor may be different from the P_D1_–P_D2_ pair. Tamura et al. (2020) have suggested that Chl_D1_ serves as the initial electron donor based on the following findings: (1) the low electronic coupling between P_D1_ and P_D2_ (approximately 10 meV) compared to the strong coupling between bacteriochlorophylls P_L_ and P_M_ (approximately 110 meV) that form a special pair in purple bacterial photosynthetic reaction centers, indicating that P_D1_ and P_D2_ do not form a special pair; (2) the low cite energy of Chl_D1_; and (3) the stabilization of Chl_D1_^•+^ by residues involved in water oxidation, implying the association between this charge separation mechanism and the water-splitting ability. The primary processes of charge separation is yet to be fully understood (Sirohiwal et al. 2020, Yoneda et al. 2022, Nguyen et al. 2023, Jha et al. 2024). Two main charge separation mechanisms have been proposed; the P_D1_ pathway: [P_D1_P_D2_]^*^ → P_D2_^+^P_D1_^−^ → P_D1_^+^Chl_D1_^−^ → P_D1_^+^Pheo_D1_^−^, and the Chl_D1_ pathway: [Chl_D1_Pheo_D1_]^*^ → Chl_D1_^+^Pheo_D1_^−^ → P_D1_^+^Pheo_D1_^−^. A recent study proposed a pathway in which Chl_D1_ and P_D1_ act in concert as the primary electron donor: [Chl_D1_Pheo_D1_P]^*^ → [Chl_D1_P_D1_]^+^Pheo_D1_^−^ → P_D1_^+^Pheo_D1_^−^ (Nguyen et al. 2023). ‘P680’ is currently more often used pointing to the four chlorophylls: P_D1_, P_D2_, Chl_D1_, and Chl_D2_ (Shevela et al. 2023).

Electron transfer from Pheo_D1_ to Q_A_ has been suggested to be facilitated by a nearby Trp residue (D2-Trp253) through superexchange electron transfer (Saito et al. 2024). In between Q_A_ and Q_B_ lies a non-heme iron ligated by four His residues and a bicarbonate ion (Shevela et al. 2012) binding to the stromal/cytosolic side of the non-heme iron (Müh and Zouni 2013). Electron transfer from Q_A_ to Q_B_, protonation of reduced Q_B_, and release of Q_B_H_2_ from the Q_B_-site are facilitated by several residues nearby, including D1-His215, D1-Ser264, and D1-His252 (Ishikita and Knapp 2005a, Saito et al. 2013, Sugo et al. 2022). The bicarbonate ligand could be involved in photoprotection by controlling *E*_m_ (Q_A_/Q_A_^−^) (Brinkert et al. 2016), in facilitating electron transfer from Q_A_ to Q_B_ (Eaton-Rye and Govindjee 1988), and/or in the exchange of Q_B_H_2_ (Sedoud et al. 2011). Q_B_H_2_ exits the Q_B_-site through the PQ exchange channels running along the sides of Cyt *b*_559_ (Van Eerden et al. 2017). Additional quinone binding sites (Q_C_ and Q_D_) are occasionally observed in these channels (Guskov et al. 2009, Kamada et al. 2023). The channels and/or Q_B_-site are accessible by various artificial quinones (Kamada et al. 2023) and quinone-site binding inhibitors (Imaizumi et al. 2025b); the Q_B_-site is one of the typical targets of herbicides inhibiting photosynthesis (Twitty and Dayan 2024).

## Water oxidation, water channels, and chloride ions

The unique water oxidation reaction of PSII is catalyzed by the Mn_4_CaO_5_ cluster in the oxygen-evolving center (OEC) (Shen 2015, Yano et al. 2024) (Fig. 1f). The cluster consists of four Mn ions, one Ca^2+^ ion, and five oxygen atoms forming a structure resembling a ‘distorted chair’ (Umena et al. 2011, Suga et al. 2015). It is ligated by six amino acid residues of D1 (D1-Asp170, Glu189, His332, Glu333, Asp342, Ala344) and one amino acid residue of CP43 (CP43-Glu354). In addition, the Mn_4_CaO_5_ cluster has four water molecules (W1 to W4) as ligands; W1 and W2 at Mn4, and W3 and W4 at Ca^2+^. Several water-filled channels within PSII connect the lumen with the Mn_4_CaO_5_ cluster, buried deep within the PSII complex (Hussein et al. 2023). These channels can serve as pathways for the inlet of substrate water molecules and/or the release of produced protons. Chloride ions (Cl^−^) are required for the water oxidation reaction, and two Cl^−^ ions (Cl-1 and Cl-2) bind nearby the Mn_4_CaO_5_ cluster (Imaizumi and Ifuku 2022) (Fig. 1d). The Cl-1 binding site is surrounded by the amino group of D2-Lys317, the backbone nitrogen of D1-Glu333, and the side chain of D1-Asn181, whereas the Cl-2 binding site is surrounded by the backbone nitrogens of D1-Asn338, D1-Phe339, and CP43-Glu354 (Murray et al. 2008, Kawakami et al. 2009, Umena et al. 2011).

Water oxidation is catalyzed through a reaction cycle of S*_i_* states (*i* = 0–4) involving four photochemical steps, as described by Kok et al. (1970), inspired by the findings by Joliot et al. (1969) (Fig. 1f). Following the light-induced charge separation, one electron is extracted from the Mn_4_CaO_5_ cluster to reduce P680^+^ via Y_Z_, and the Mn_4_CaO_5_ cluster advances from the S*_i_* (*i* = 0–3) state to the S_*i* + 1_ state, storing oxidizing equivalents. When the Mn_4_CaO_5_ cluster in the S_3_ state is oxidized, it forms a transient S_4_ state, molecular oxygen (O_2_) is formed and released, and the Mn_4_CaO_5_ cluster returns to the S_0_ state. In this way, the Mn_4_CaO_5_ cluster couples the four-electron oxidation of two water molecules with the one-electron photochemical reaction at P680, resulting in the oxidation of two water molecules to one molecular oxygen, four protons, and four electrons in each cycle. The most reduced S_0_ state is slowly oxidized to S_1_ by Y_D_ (D2-Tyr160) in the dark, making S_1_ the dark-stable state (Styring and Rutherford 1987). The mechanism of water oxidation has been intensively reviewed (Shen 2015, Lubitz et al. 2019, Shevela et al. 2023, Yano et al. 2024).

Controlled delivery of substrate water molecules to the Mn_4_CaO_5_ cluster and release of protons from the Mn_4_CaO_5_ cluster are essential for efficient water oxidation. In the S-state cycle, one water molecule is delivered during the S_2_ → S_3_ transition and another is delivered during the S_3_ → [S_4_] → S_0_ transition after releasing molecular oxygen. The release of four protons during the cycle usually occurs with the pattern of 1:0:1:2 for the S_0_ → S_1_ → S_2_ → S_3_ → S_0_ transitions. Three major conserved water channels are thought to be important for water inlet and proton egress: the O1 channel, the O4 channel (or O4 water chain [Saito et al. 2015; Ishikita and Saito 2025]), and the Cl-1 channel (Sakashita et al. 2017, Hussein et al. 2023). These channels have several alternative names (e.g. ‘large channel’ for the O1 channel, ‘narrow channel’ for the O4 channel, and ‘broad channel’ or ‘E65/E312 channel’ for the Cl-1 channel) as recently summarized by Hussein et al. (2023). While there remains some controversy, extensive experimental and computational studies have provided crucial insights into the pathways of water intake and proton release during different S-state transitions (Yano et al. 2024, Ishikita and Saito 2025). Water intake during the S_2_ → S_3_ transition likely occurs through the O1 channel (Suga et al. 2019, Ibrahim et al. 2020, Hussein et al. 2021, Okamoto et al. 2021, Li et al. 2024a), and this may also be the case during the S_3_ → S_0_ transition (Bhowmick et al. 2023). Proton release during the S_0_ → S_1_ transition is suggested to occur through the O4 channel (Saito et al. 2015, Takaoka et al. 2016, Shimizu et al. 2018, Sakashita et al. 2020). The Cl-1 channel is likely the pathway for proton release during the S_2_ → S_3_ and S_3_ → S_0_ transitions (Ishikita et al. 2006, Service et al. 2010, Suzuki et al. 2013, Hussein et al. 2021, Okamoto et al. 2021, Shimada et al. 2022, Allgöwer et al. 2024, Noguchi 2024, Li et al. 2024a, Flesher et al. 2025).

Cl^−^ ions are also essential for water oxidation. Of the two Cl^−^ ions bound near the Mn_4_CaO_5_ cluster, the functions and importance of Cl-1 have been revealed through intensive studies, while little is known about those of Cl-2 (Imaizumi and Ifuku 2022). Cl-1 is directly involved in the Cl-1 channel and has a critical role in maintaining the hydrogen-bond network in the channel. In the absence of Cl-1, D2-Lys317 and D1-Asp61 form a salt bridge that inhibits proton transfer through this channel (Rivalta et al. 2011, Mandal et al. 2022). In addition, loss of charge compensation for D2-Lys317 has been suggested to cause an increase in *E*_m_ (S_2_/S_3_), making the electron transfer from S_2_/S_3_ to Y_Z_ an uphill electron transfer pathway; Cl-1 is required to push away the electron of S_2_ towards Y_Z_ and stabilize the oxidized S_3_ state (Mandal et al. 2022). In D2-K317A mutant *Synechocystis* sp. PCC 6803, the oxygen-evolving activity of PSII was reduced, but the Cl^−^ requirement seemed to be lost, suggesting that a major role of Cl-1 is dependent on D2-Lys317 (Pokhrel et al. 2013, Mandal et al. 2022, Flesher et al. 2025). Cl-1 also regulates the deprotonation of the ligand water molecules in the Mn_4_CaO_5_ cluster. During the S_2_ → S_3_ transition, Cl-1 inhibits deprotonation of W2 (Saito et al. 2020), thereby facilitating proton release from W1 via D1-Asp61 (Kawashima et al. 2018). As for Cl-2, its roles and even its necessity have remained unclear. However, a recent study on PsbP-Loop 4 near the Cl-2 site in green plant PSII suggested that Cl-2 can be important for a high oxygen-evolving activity of PSII (Imaizumi et al. 2022). Revisiting reports (Okumura et al. 2001, 2007) before the identification of the Cl^−^ binding sites (Murray et al. 2008, Kawakami et al. 2009), Cl-2 also seems to be important for the oxygen-evolving activity in cyanobacterial and red-lineage PSII (Imaizumi et al. 2022). Cl-2 is closely related to the O4 channel, and may have a role in the formation of an ordered water chain, required for efficient proton transfer (Imaizumi et al. 2022, Imaizumi and Ifuku 2022). Interestingly, although many red-lineage PSII structures from different red algae (Ago et al. 2016, You et al. 2023) and various red-lineage algae (Nagao et al. 2019, Pi et al. 2019, Nagao et al. 2022, Feng et al. 2023, Zhao et al. 2023, Mao et al. 2024, Si et al. 2024, Zhang et al. 2024a) have been reported, Cl-1 has not been modeled in any of these structures except for one (PDB ID: 8XLP) lacking the Mn_4_CaO_5_ cluster and extrinsic subunits, whereas Cl-2 has been modeled in most of these. Whether or not Cl-1 is present in red-lineage PSII requires further confirmation.

## Extrinsic Subunits

### Compositions of extrinsic subunits

**Fig. 2:**
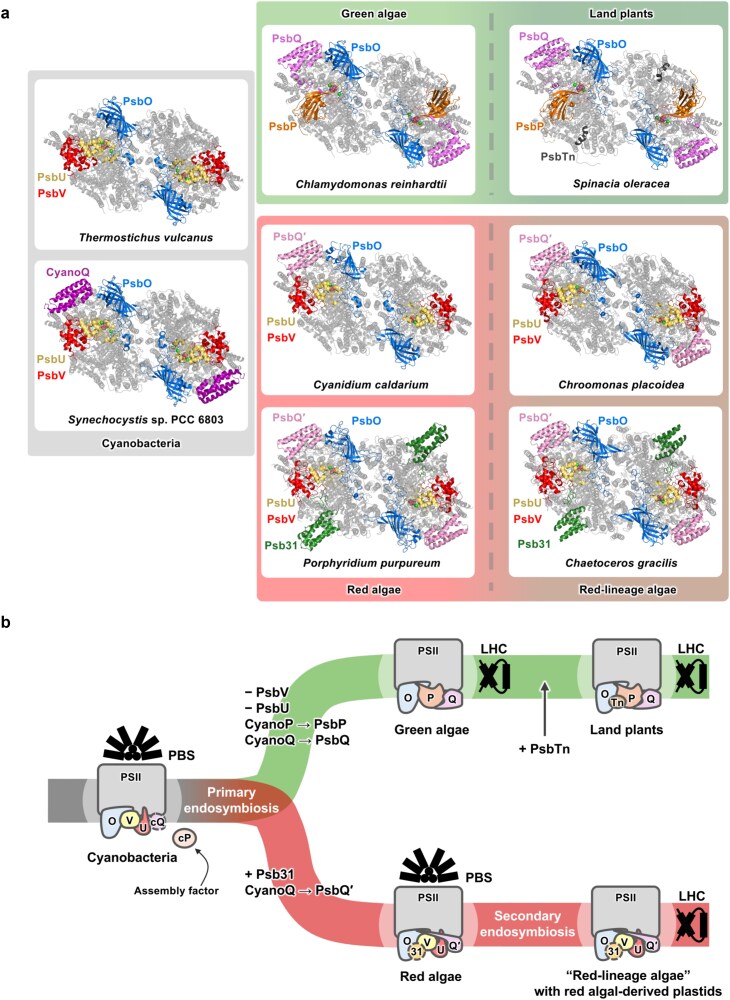
Binding sites and the evolutionary changes in the compositions of the extrinsic subunits of PSII. (a) Binding sites of extrinsic subunits in PSII from various species. The lumenal side of the structures of dimeric PSII cores from a thermophilic cyanobacterium *Thermostichus vulcanus* (PDB ID: 3WU2), a mesophilic cyanobacterium *Synechocystis* sp. PCC 6803 (PDB ID: 7N8O), a green alga *Chlamydomonas reinhardtii* (PDB ID: 6KAC), a land plant *Spinacia oleracea* (PDB ID: 8Z9D), red algae *Cyanidium caldarium* (PDB ID: 4YUU) and *Porphyridium purpureum* (PDB ID: 7Y5E), a cryptophyte (red-lineage alga) *Chroomonas placoidea* (PDB ID: 8XR6), and a diatom (red-lineage alga) *Chaetoceros gracilis* (PDB ID: 6JLU) are shown. The extrinsic subunits are colored as follows: PsbO (blue), PsbV (red), PsbU (yellow), PsbP (orange), CyanoQ (purple), PsbQ (pink), PsbQ′ (light pink), Psb31 (green), PsbTn (dark gray). The Mn_4_CaO_5_ cluster is shown as a cluster of red, purple, and orange spheres, and the Cl^−^ ions are shown as green spheres. (b) Schematic illustration of the evolutionary changes in the compositions of the PSII extrinsic subunits. Cyanobacteria possess PsbO (O), PsbV (V), PsbU (U), and, at least in some species, CyanoQ (cQ) as PSII extrinsic subunits. They also possess an assembly factor, CyanoP (cP). Primary endosymbiosis led to the development of the green lineage and red lineage. In the green lineage, PsbV and PsbU were lost, and PsbP (P) and PsbQ (Q) evolved from CyanoP and CyanoQ, respectively. Green algal PSII binds PsbO, PsbP, and PsbQ, whereas land plants further acquired PsbTn (Tn). In the red lineage, PsbQ′ (Q′) developed from CyanoQ and replaced it as an extrinsic subunit, and a new extrinsic subunit, Psb31 (31), was acquired at an early stage before red algae diversified. Psb31 is retained in some red algae and red-lineage algae, while others have lost it. The usage of phycobilisomes (PBSs) or light-harvesting complexes (LHCs) in the different species is indicated by illustrations on top of or on the side of PSII.

Membrane-extrinsic subunits bind to the lumenal side of PSII, surrounding the Mn_4_CaO_5_ cluster. While the structure of the central membrane embedded part of PSII is mostly well-conserved among diverse oxyphototrophs, the compositions of the extrinsic subunits have largely changed during evolution (Fig. 2). Cyanobacterial PSII has a set of three or four extrinsic subunits: PsbO, PsbV, PsbU, and, additionally, CyanoQ. Red-lineage PSII (including red algal PSII and red-lineage algal PSII) binds four or five extrinsic subunits: PsbO, PsbV, PsbU, PsbQ′, and in some species, Psb31. Green plant PSII (including green algal PSII and land plant PSII) binds three or four extrinsic subunits: PsbO, PsbP, PsbQ, and, in many land plants, PsbTn. PsbR had previously been proposed as another extrinsic subunit of green plant PSII, but recent studies have revealed that it is an intrinsic subunit with a long stromal loop region and only a few residues exposed to the lumen (Shan et al. 2024). Little is known about PSII in glaucophytes, but PSII of a glaucophyte, *Cyanophora paradoxa*, seems to bind PsbO, PsbV, and PsbU, similar to cyanobacterial PSII (Shibata et al. 2001, Enami et al. 2005). The extrinsic subunits are highly charged, and differences in charge distribution on the lumenal surface (Supplementary Fig. S2) could affect the energetics of proton/electron transfer. Structures and functions of the extrinsic subunits have been reviewed previously (Bricker et al. 2012, Roose et al. 2016, Ifuku and Nagao 2021). Here, we provide an updated comprehensive summary on the functions of the extrinsic subunits of PSII. Unique viewpoints and insights from the most recent findings are discussed, aiming to advance our current understanding of the extrinsic subunits of PSII.

### Individual characteristics

#### PsbO

PsbO, also referred to as OEC33 or the 33 kDa extrinsic protein (Åkerlund and Jansson 1981, Yamamoto et al. 1981), is the only extrinsic subunit that is commonly observed in PSII in all oxyphototrophs (De Las Rivas et al. 2004, Enami et al. 2005) (Fig. 2). This subunit is also known as the manganese-stabilizing protein, as it has essential roles in stabilizing the Mn_4_CaO_5_ cluster (Miyao and Murata 1984, Ono and Inoue 1984, Ghanotakis et al. 1984a). PsbO consists of two major domains: a flexible head domain (mostly a large loop) and a β-barrel domain (De Las Rivas and Barber 2004). A pair of conserved Cys residues form a disulfide bond, suggested to be important for the structural stability and in vivo accumulation of PsbO (Tanaka and Wada 1988, Burnap et al. 1994, Karamoko et al. 2011). PsbO mainly interacts with the lumenal regions of the D1, D2, CP47, and CP43 subunits, as well as with some other extrinsic subunits. Within PSII dimers, it also interacts with CP47 in the adjacent monomer. The N-terminal region of PsbO likely has a role in regulating the specific binding of PsbO to PSII (Eaton-Rye and Murata 1989, Seidler et al. 1996, Popelkova et al. 2002, Popelkova and Yocum 2011).

A major role of PsbO is to optimize and stabilize the structure of the OEC. Release-reconstitution experiments have shown that the absence of PsbO leads to a large decrease in oxygen-evolving activity and destabilized binding of Mn (Miyao and Murata 1984, Ono and Inoue 1984, Kuwabara et al. 1985). Light-induced Fourier transform infrared (FTIR) difference spectroscopy, used to study the structural changes in the OEC during the process of water oxidation (Noguchi 2015, Ifuku and Noguchi 2016), has revealed that PsbO is crucial for the proper protein conformation in the OEC in cyanobacterial PSII (Nagao et al. 2015), although in land plant PSII, PsbP (Tomita et al. 2009), and in red algal PSII, PsbV (and PsbU) (Uno et al. 2013), are also required. A study on the protein dynamics of the PSII lumenal region using high-speed atomic force spectroscopy suggested that PsbO suppresses the conformational fluctuation of the CP43 lumenal domain and stabilizes the OEC structure (Tokano et al. 2020). Furthermore, cryo-EM structures of PSII lacking the OEC have implied that PsbO may affect the conformations of the C-terminal region of D1 (Gisriel et al. 2020, Zabret et al. 2021).

The importance of PsbO has also been revealed in knockout mutants, although the extent of the effects of PsbO depletion in vivo depends on the species. ΔPsbO (PsbO-deficient) mutants of the cyanobacterium *Synechocystis* showed decreased oxygen-evolving activity, partially retarded photoautotrophic growth, increased Ca^2+^ requirement for photoautotrophic growth, and enhanced susceptibility to photoinhibition (Burnap and Sherman 1991, Philbrick et al. 1991, Burnap et al. 1992, Morgan et al. 1998). ΔPsbO mutants of the green alga *Chlamydomonas reinhardtii* did not show any oxygen-evolving activity and were incapable of photoautotrophic growth (Mayfield et al. 1987a). *Arabidopsis thaliana* possesses two PsbO isoforms, PsbO1 and PsbO2, with PsbO1 being the major isoform (Murakami et al. 2005). Mutants lacking only PsbO1 showed growth retardation and lower oxygen-evolving activity (Murakami et al. 2002), and were unable to efficiently use Ca^2+^ for water oxidation (Bricker and Frankel 2008). The *psbo1 psbo2* double knockout mutant (Suorsa et al. 2016) and PsbO-RNAi plants with low PsbO expression could not grow photoautotrophically (Yi et al. 2005). Cyanobacterial ΔPsbO mutants showed increased stabilization of the S_2_ and S_3_ states, and retardation of the S_3_ → [S_4_] → S_0_ transition (Burnap et al. 1992), and *A. thaliana psbo1* mutants have shown similar impairments in S-state transitions (Liu et al. 2007). These effects on the S-state cycle of PSII are in accordance with the results from in vitro studies (Ono and Inoue 1985, Miyao et al. 1987).

PSII structures have revealed that part of the PsbO head domain is inserted near the OEC. This includes the highly conserved residue PsbO-Asp158 (*Thermosynechococcus* numbering; Asp157 in spinach and Asp159 in *Synechocystis*), located near Cl-1 and closely related to the Cl-1 channel (Popelkova and Yocum 2011, Russell and Vinyard 2024). Mutations at this site led to low oxygen-evolving activity and stable S_2_ and S_3_ states, and have affected the proper binding of PsbO to PSII (Burnap et al. 1994, Roose et al. 2010a, Zhu et al. 2020). The backbone amides of PsbO-Asp158 and PsbO-Leu157 form hydrogen bonds with the side chains of D1-Arg334 and D1-Asn335, and these two residues interact with D1-Glu65 and D2-Glu312 (Guerra et al. 2018, Russell and Vinyard 2024). This D1-E65/D2-E312 dyad acts as a gate for proton transfer through the Cl-1 channel, and D1-Arg334 and D1-Asn335 are involved in the opening/closing of the gate (Hussein et al. 2021, Bhowmick et al. 2023, Noguchi 2024). In addition to the Cl-1 channel (Ishikita et al. 2006, Ifuku and Nagao 2021, Hussein et al. 2023, Allgöwer et al. 2024), PsbO also partially affects the O4 channel (Takaoka et al. 2016, Hussein et al. 2023).

**Fig. 3:**
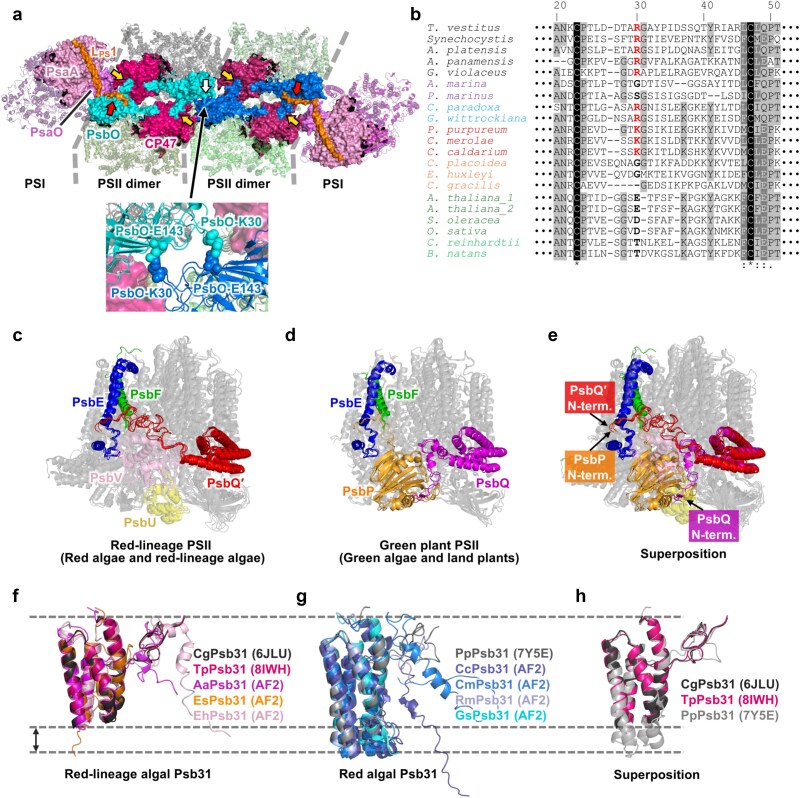
Recent insights into the structures and functions of extrinsic subunits. (a) Structural roles of PsbO in the supramolecular organization of PSII. PSII and PSI in the PBS–PSII–PSI–LHC megacomplex from a red alga *Porphyridium purpureum* (PDB ID: 7Y5E) viewed from the lumenal side with PsbO (light blue in one dimer and dark blue in the other), CP47 (red in one dimer and purple in the other), PsaA (light pink), PsaO (pink), and L_PS_1 (orange) shown as surface representation. Yellow arrows indicate inter-monomer interaction between PsbO and CP47, commonly observed in PSII from various species. Red arrows indicate interaction of PsbO with the photosystem linker protein L_PS_1. The white arrow indicates inter-dimer interaction between two PsbO subunits of adjacent PSII dimers. An enlarged view of the inter-dimer interaction site with the interacting residues shown as spheres is provided below the megacomplex structure. (b) Multiple sequence alignment of PsbO from various species conducted with MAFFT (v7.511). Only the region including the two conserved Cys residues and the Arg/Lys residue involved in inter-dimer interactions is shown. PsbO sequences from the following species were used. Five cyanobacteria that use phycobilisomes: *Thermosynechococcus vestitus* (WP_011056297.1), *Synechocystis* (multispecies) (WP_010873097.1), *Arthrospira platensis* C1 (EKD06931.1), *Anthocerotibacter panamensis* (WP_218079464.1), and *Gloeobacter violaceus* PCC 7421 (BAC91632.1). Two cyanobacteria that use other light-harvesting antennas: *Acaryochloris marina* (WP_012161181.1) and *Prochlorococcus marinus* MIT 9313 (CAI8253261.1). Two glaucophytes: *Cyanophora paradoxa* (CAH04962.1) and *Gloeochaete wittrockiana* (BAF94210.1). Three red algae: *P. purpureum* (KAA8497770.1), *Cyanidioschyzon merolae* strain 10D (XP_005536430.1), and *Cyanidium caldarium* (KAK4534118.1). Three red-lineage algae: *Chroomonas placoidea* (XCO00665.1), *Emiliania huxleyi* CCMP1516 (XP_005761454.1), and *Chaetoceros gracilis* (BAG85210.1). Land plants, a green alga, and a chlorarachniophyte: *Arabidopsis thaliana* (PsbO1: NP_201458.1; PsbO2: NP_190651.1), *Spinacia oleracea* (NP_001413402.1), *Oryza sativa* Japonica group (XP_015618593.1), *Chlamydomonas reinhardtii* (XP_001694699.1), and *Bigelowiella natans* (AAP79149.1). The Arg/Lys residue corresponding to *Porphyridium* PsbO-Lys30 is shown with bold red letters. Asterisks (*), colons (:), and dots (.) below the aligned sequences indicate identical, conserved, and semi-conserved amino acids, respectively. Identical amino acids are shown with black backgrounds, conserved amino acids matching the consensus sequence are shown with gray backgrounds, and conserved amino acids that do not match the consensus sequence, semi-conserved amino acids, and non-conserved amino acids matching the consensus sequence are shown with light gray backgrounds. (c–e) Structural comparison of the N-terminal regions of PsbP, PsbQ, and PsbQ′. (c) Superposition of red-lineage PSII structures of *P. purpureum* (PDB ID: 7Y5E), *C. gracilis* (PDB ID: 6JLU), *Thalassiosira pseudonana* (PDB ID: 8IWH), and *C. placoidea* (PDB ID: 8XR6). (d) Superposition of green plant PSII structures of *Pisum sativum* (PDB ID: 5XNL), *S. oleracea* (PDB ID: 3JCU), and *C. reinhardtii* (PDB ID: 6KAC). (e) Superposition of all PSII structures in panels c and d. (f–h) Structural comparison of red-lineage algal and red algal Psb31. Protein structure predictions of Psb31 were performed by AlphaFold2 (AF2) using ColabFold v1.5.5, and top-ranked models were used. (f) Superposition of red-lineage algal Psb31 structures: *C. gracilis* Psb31 (PDB ID: 6JLU), *T. pseudonana* Psb31 (PDB ID: 8IWH), and predicted Psb31 structures from *Aureococcus anophagefferens*, *Ectocarpus siliculosus*, and *E. huxleyi* CCMP1516. (g) Superposition of red algal Psb31 structures: *P. purpureum* Psb31 (PDB ID: 7Y5E) and predicted Psb31 structures from *C. caldarium*, *C. Merolae* strain 10D, *Rhodosorus marinus*, and *Galdieria sulphuraria*. (h) Superposition of Psb31 from red-lineage algae (*C. gracilis* [PDB ID: 6JLU] and *T. pseudonana* [PDB ID: 8IWH]) and a red alga (*P. purpureum* [PDB ID: 7Y5E]).

Other than its roles in the OEC, PsbO also seems to be involved in the supramolecular organization of PSII (Fig. 3a). While mature PSII is usually dimeric, accumulation of PSII core dimers was strongly suppressed in cyanobacterial ΔPsbO mutants (Bentley and Eaton-Rye 2008, Komenda et al. 2010). Similarly, *psbO1* and *psbO2* mutant *A. thaliana* showed decreased accumulation of dimeric PSII cores (Lundin et al. 2008). In vitro experiments using isolated cyanobacterial or green plant PSII showed that the removal of PsbO leads to destabilization of PSII core dimers (Dekker et al. 1988, Boekema et al. 2000). Inter-monomer interaction within PSII dimers has been observed between PsbO of one monomer and CP47 of the other, and this is thought to play a role in stabilizing the PSII core dimer at the monomer–monomer interface on the lumenal side (De Las Rivas and Barber 2004, Li et al. 2024b). Cryo-EM structures of monomeric PSII support this view; the PsbO structure became flexible in PSII monomers, supposedly due to the loss of interaction with CP47 from the other monomer (Yu et al. 2021), and the conserved β-sheet structure of CP47, which would interact with the adjacent monomer’s PsbO in PSII dimers, was lost in a PSII monomer lacking PsbO and other extrinsic subunits (Gisriel et al. 2020).

PsbO may also be involved in stabilizing PSII core dimer–dimer interactions in cyanobacteria and red algae. Recently, *in situ* cryo-ET structures of a PBS (phycobilisome)–PSII supercomplex from the cyanobacterium *Arthrospira* sp. FACHB-439 (Zhang et al. 2024b), and PBS–PSII supercomplexes (Li et al. 2021a) and PBS–PSII–PSI–LHC (light-harvesting complex) megacomplexes (You et al. 2023) from the red alga *Porphyridium purpureum* have revealed the detailed association between adjacent PSII core dimers within linear PSII dimer arrays. On the lumenal side, PsbO is responsible for the PSII dimer–dimer connection through interaction with PsbO of the adjacent PSII dimer (Fig. 3a). In red algal PSII, salt bridges were formed between PsbO-Lys30 of a PSII dimer and PsbO-Glu143 of the adjacent PSII dimer (You et al. 2023). In cyanobacterial PSII, PsbO-Arg55 (corresponding to PsbO-Lys30 in *P. purpureum*) formed a salt bridge with PsbO-Glu241 (not corresponding to *P. purpureum* PsbO-Glu143) of the adjacent PSII dimer, and several additional hydrogen bonds were also observed (Zhang et al. 2024b). Meanwhile, such interaction is unlikely to occur in red-lineage algal or green plant PSII, which use membrane-embedded LHCs as PSII antennas, as the above PSII dimer–dimer interface corresponds to the PSII–LHCII interface. We compared the amino acid sequences of PsbO from various species, including red-lineage algae, green algae, and land plants that use LHCs, as well as cyanobacteria, glaucophytes, and red algae that generally use phycobilisomes (Fig. 3b). The Arg/Lys residue (corresponding to PsbO-Lys30 in *P. purpureum*) was highly conserved only in cyanobacteria, glaucophytes, and red algae which use phycobilisomes, and was not observed in red-lineage algae, green algae, or land plants which use LHCs, nor in atypical cyanobacteria (*Acaryochloris marina* and *Prochlorococcus marinus*) that use membrane-intrinsic prochlorophyte chlorophyll-binding (Pcb) proteins as their peripheral light-harvesting antennas (Bibby et al. 2003, Shen et al. 2024). The formation of an inter-dimer salt bridge between the specific Arg/Lys residue of PsbO (corresponding to PsbO-Lys30 in *P. purpureum*) of one dimer and a Glu residue of PsbO of the adjacent dimer may be a conserved feature stabilizing the PSII dimer–dimer association, facilitating the formation of a tightly packed linear PSII dimer array in species that use PBSs as their PSII light-harvesting antennas.

In addition, in the red algal PBS–PSII–PSI–LHC megacomplexes, PSI is bound to each end of the PSII linear array, and PsbO was also found to be involved in the interaction between PSII and PSI (You et al. 2023). At the PSII–PSI interaction interface, a lumenal linker protein denoted photosystem linker protein 1 (L_PS_1) was found to tightly bind to PsbO with its N-terminal region and interact with PSI subunits PsaA and PsaO with its C-terminal helix (Fig. 3a).

#### PsbV

PsbV, also known as Cyt *c*_550_ (Holton and Myers 1963), is an extrinsic subunit found in cyanobacterial (Shen et al. 1992), red algal (Enami et al. 1995), and red-lineage algal PSII (Enami et al. 2005, Nagao et al. 2007) (Fig. 2). It binds to PSII near the lumenal regions of D1 and CP43. Although it is a cytochrome, it remains unclear whether it has any redox functions in PSII (Guerrero et al. 2011, Khorobrykh et al. 2018). It is interesting to note that PSII-bound PsbV and unbound PsbV exhibit a different redox potential due to changes in protein backbone orientations near the heme group upon binding to PSII (Ishikita and Knapp 2005b).

PsbV has a critical role in Cl^−^ and Ca^2+^ retention in the OEC. Cyanobacterial ΔPsbV mutants cannot grow autotrophically in medium depleted of either Cl^−^ or Ca^2+^, although they can grow at partially reduced rates in the presence of both Cl^−^ and Ca^2+^ (Morgan et al. 1998, Shen et al. 1998, Katoh et al. 2001). In vitro release-reconstitution experiments support that PsbV has an important role in Cl^−^ and Ca^2+^ retention in cyanobacterial, red algal, and red-lineage algal PSII (Shen and Inoue 1993, Enami et al. 1998, Nagao et al. 2010). FTIR analyses of cyanobacterial (Nagao et al. 2015) and red algal PSII (Uno et al. 2013) have shown that PsbV is required for the proper protein conformation in the OEC. PsbV is also important for the binding of PsbU to PSII (Shen and Inoue 1993, Enami et al. 1998, Nagao et al. 2010). Its importance for PsbU binding could slightly differ among different species, including *Synechocystis* and *Thermostichus vulcanus* (Eaton-Rye et al. 2003). Additionally, PsbV is involved in the O1 channel (or O1-PsbU/PsbV channel) (Sakashita et al. 2017, Hussein et al. 2023), and could indirectly affect the Cl-1 channel (Ifuku and Nagao 2021).

#### PsbU

PsbU is a 12 kDa protein, initially found as an 9 kDa protein (Stewart et al. 1985, Rolfe and Bendall 1989), present in cyanobacterial (Shen et al. 1992), red algal (Enami et al. 1995), and red-lineage algal PSII (Enami et al. 2005, Nagao et al. 2007) (Fig. 2). It binds to PSII at the lumenal side of CP43, CP47, and PsbV, with its C-terminal region inserted near the C-terminal regions of D1 and D2. Similar to PsbV, a major role of PsbU is to support the retention of Cl^−^ and Ca^2+^ ions in the OEC. Normally, cyanobacterial ΔPsbU mutants can grow photoautotrophically at similar rates as the wild-type with partially lower oxygen-evolving activity, but in growth medium depleted of either Cl^−^ or Ca^2+^, PsbU-deficiency leads to slightly lower growth rates than the wild-type (Shen et al. 1997, Inoue-Kashino et al. 2005, Summerfield et al. 2005). When both Cl^−^ and Ca^2+^ are depleted, ΔPsbU mutants cannot grow photoautotrophically (Inoue-Kashino et al. 2005). The roles of PsbU in supporting Cl^−^ and Ca^2+^ retention are also suggested from release-reconstitution experiments using cyanobacterial, red algal, and red-lineage algal PSII (Shen and Inoue 1993; Enami et al. 1998; Nagao et al. 2010). Together with PsbO and PsbV, PsbU has a role in maintaining the proper protein conformation in the OEC, as suggested from FTIR analyses of cyanobacterial (Nagao et al. 2015) and red algal PSII (Uno et al. 2013). PsbU may also have some other additional roles, such as possible transmembrane effects on energy transfer from PBS to PSII (Veerman et al. 2005).

While efficient binding of PsbU to PSII requires the presence of PsbO and PsbV, PsbU supports the functional binding of PsbV to PSII (Shen and Inoue 1993; Enami et al. 1998; Nagao et al. 2010). Therefore, the effects of PsbU on ion retention and the protein conformation of the OEC can be due to both direct functions of PsbU and enhanced functional binding of PsbV to PSII. However, at least the C-terminal region of PsbU seems to have a direct role in supporting Cl^−^ retention. Release-reconstitution experiments using red algal PSII have revealed that the residue PsbU-Tyr92 (corresponding to Tyr103 in cyanobacterial PsbU) in the C-terminal region has a critical role in Cl^−^ retention, while having little effect on the binding of PsbV or PsbU to PSII (Okumura et al. 2001, 2007). Although the Cl^−^ binding sites had not been determined at that time, interaction between PsbU-Tyr103 and D1-Pro340 was proposed to be important for Cl^−^ retention. It is now known that Cl-2 binds adjacent to D1-Asn338 and D1-Phe339 (and CP43-Glu354), and PsbU-Tyr103 is likely important for the retention of Cl-2 (Imaizumi et al. 2022, Imaizumi and Ifuku 2022). PsbU is also suggested to be involved in the O4 channel (O4-PsbU channel) (Saito et al. 2015, Takaoka et al. 2016, Sakashita et al. 2017, Hussein et al. 2023) and the O1 channel (O1-PsbU/PsbV channel) (Sakashita et al. 2017, Hussein et al. 2023, Li et al. 2024a), and may have some effects on the Cl-1 channel (Ifuku and Nagao 2021, Hussein et al. 2023).

#### PsbP

PsbP, also designated OEC23 due to its apparent molecular mass of 23 kDa, is an extrinsic subunit unique to green plant PSII (Åkerlund and Jansson 1981,Yamamoto et al. 1981, Åkerlund et al. 1982) (Fig. 2). Intensive studies have elucidated the important roles of PsbP for water oxidation by PSII. In land plants, PsbP is essential for photoautotrophy. Knockdown of PsbP by RNA interference (RNAi) leads to severe growth defects, low PSII activity, and impaired accumulation of PSII-LHCII supercomplexes, resulting in disordered grana stacks (Ifuku et al. 2005, Yi et al. 2007, Ido et al. 2009). PsbP-knockout mutants show a seedling-lethal phenotype (Allahverdiyeva et al. 2013). Chlorotic symptoms of various viral infections seem to be induced by decreased PsbP accumulation as well. PsbP has been reported to be affected by the infection of various viruses such as *Cucumber mosaic virus* (CMV), *Tobacco mosaic virus* (TMV), *Alfalfa mosaic virus* (AMV), *Rice stripe virus* (RSV), *Pepper mild mottle virus* (PMMoV), *Paprika mild mottle virus* (PaMMoV), and a gemnivirus betasatelite (radish leaf curl betasatellite; RaLCB); infection of most of these viruses led to decreased expression and/or accumulation of PsbP, and interaction of PsbP with the coat protein of AMV or with the disease-specific protein of RSV in the cytosol prevented cytosolically synthesized PsbP from entering the chloroplast (Takahashi et al. 1991, Rahoutei et al. 2000, Pérez-Bueno et al. 2004, Sui et al. 2006, Balasubramaniam et al. 2014, Kong et al. 2014, Gnanasekaran et al. 2019). The importance of PsbP has also been studied in green algal PsbP-deficient mutants; *C. reinhardtii* mutant strains lacking PsbP (BF25 and FUD39) (Rochaix 1987, Mayfield et al. 1987b, de Vitry et al. 1989) show photoautotrophic growth defects and low PSII activity.

PsbP binds to PSII at a similar site as PsbV and PsbU in cyanobacterial and red-lineage PSII (Wei et al. 2016, Sheng et al. 2019, Imaizumi and Ifuku 2022) (Fig. 2a and Fig. 3c–e), and the major functions of PsbP resemble those of PsbV and PsbU as well. Release-reconstitution experiments have revealed that PsbP critically supports the retention of the Cl^−^ (Andersson et al. 1984, Miyao and Murata 1985) and Ca^2+^ (Ghanotakis et al. 1984b) ions in the OEC. Various mutations in PsbP decrease its ion (Cl^−^ and/or Ca^2+^) retention ability (Imaizumi and Ifuku 2022), whereas one mutation (PsbP-D139N) increased its Cl^−^ (but not Ca^2+^) retention ability (Imaizumi et al. 2022). Ion retention in the OEC by PsbP has recently been summarized in detail (Imaizumi and Ifuku 2022). FTIR analyses have shown that PsbP is required for inducing proper conformational changes in the OEC (Tomita et al. 2009), including the protein conformation around the Cl^−^ binding sites (Kondo and Noguchi 2018). Various mutations in PsbP alter or perturb these conformational changes (Ifuku and Noguchi 2016). PsbP also protects the OEC from exogenous reductants (Ghanotakis et al. 1984c). Recent studies have shown the involvement of PsbP in water channels as well. The O1 (O1-PsbU/PsbV) channel proceeding towards PsbV and PsbU and the O4 (O4-PsbU) channel proceeding towards PsbU in cyanobacterial PSII are structurally conserved as the O1-PsbP channel and the O4-PsbP channel, respectively, both proceeding towards PsbP (Sakashita et al. 2017, Hussein et al. 2023).

In addition to its crucial roles in water oxidation, PsbP also has transmembrane effects on the acceptor side of PSII. The N-terminal region of PsbP interacts with the well-conserved regions in the lumenal domain of Cyt *b*_559_ (Imaizumi et al. 2025a) (Fig. 3d). Depletion of PsbP leads to the conversion of Cyt *b*_559_ from its high potential (HP) form to its intermediate potential (IP) or low potential (LP) forms (Ghanotakis et al. 1986, Thompson et al. 1989), and the HP form recovers by the reconstitution of a peptide consisting of the first 15 N-terminal residues of PsbP (Nishimura et al. 2016). This indicates that, although the redox-active heme of Cyt *b*_559_ is located close to the stromal side of the thylakoid membrane, the interaction between the N-terminal region of PsbP and Cyt *b*_559_ at the lumenal side is important for maintaining the HP form of Cyt *b*_559_ in green plant PSII. PsbP depletion also causes suppression of electron transfer from Q_A_^−^ to Q_B_ and a shift in the redox potential of Q_A_^−^/Q_A_, although Q_A_ and Q_B_ are also located near the stromal side of the thylakoid membrane (Yi et al. 2007, Roose et al. 2010b, Semin et al. 2018, Kato and Noguchi 2021). These transmembrane effects of PsbP can be related to photoprotective roles (Nishimura et al. 2016, Kato and Noguchi 2021, 2022), and PsbP has been proposed to balance the oxidizing side and reducing side of PSII (Ifuku and Nagao 2021, Imaizumi and Ifuku 2022).

#### PsbQ

PsbQ, which used to be called OEC16, OEC17, or OEC18, due to its apparent molecular mass of 16–18 kDa, is another extrinsic subunit unique to green plant PSII (Yamamoto et al. 1981, Åkerlund et al. 1982) (Fig. 2). Its structure consists of a long flexible N-terminal loop and a four-helix bundle core. The four-helix bundle core binds to the lumenal surface of CP43, and the N-terminal loop attaches along the lumenal surface of PsbP (Fig. 3d). Although, phylogenetically, green algal PsbQ is closer to red-lineage PsbQ′ than land plant PsbQ (Yabuta et al. 2010), structurally, green algal PsbQ associates to PSII in a similar manner as land plant PsbQ does (Wei et al. 2016, Sheng et al. 2019, Shan et al. 2024) (Fig. 3c–e). Moreover, green algal and land plant PsbQ both possess the well-conserved (Gln/Glu)25–Asp28 region (numbered based on spinach PsbQ) (Imaizumi and Ifuku 2022), whereas green algal PsbQ does not possess the well-conserved N-terminal sequence characteristic of PsbQ′ (Imaizumi et al. 2025a).

In vitro release-reconstitution experiments have shown that PsbQ supports the Cl^−^ retention by PsbP, especially under very low Cl^−^ concentrations in both land plant PSII (Akabori et al. 1984, Imaoka et al. 1984, Miyao and Murata 1985) and green algal PSII (Suzuki et al. 2003). In the presence of mutant PsbP with functional defects, PsbQ further supports the functions of PsbP, most likely by assisting the functional binding of PsbP to PSII. For example, PsbQ largely helps the binding of the N-terminally truncated Δ15-PsbP (lacking the first 15 N-terminal residues) to PSII, partially restores the Cl^−^ and Ca^2+^ retention ability of Δ15-, Δ19-, and H144A-PsbP, and partially recovers the proper conformational changes in the OEC in the presence of Δ15- or H144A-PsbP (Ifuku and Sato 2002, Kakiuchi et al. 2012). A study using N-terminally truncated PsbQ, lacking the first 12 N-terminal residues, showed that the N-terminal region of PsbQ is important for the binding of PsbQ to PSII, and essential for supporting Cl^−^ retention under low Cl^−^ concentrations (Kuwabara et al. 1986). The N-terminal region of PsbQ is closely associated with PsbP, and this interaction is likely to be important for supporting the binding and functions of PsbP (Imaizumi and Ifuku 2022). PsbQ may affect the O1 water channel (Sakashita et al. 2017; Hussein et al. 2023), and this might also be related to Cl^−^ retention (Imaizumi and Ifuku 2022).

In contrast to the roles observed in the presence of mutant PsbP, in the presence of wild-type PsbP, PsbQ has little effect on the binding of PsbP to PSII (Kakiuchi et al. 2012), the retention of Ca^2+^ in the OEC (Suzuki et al. 2003), or the conformational changes in the OEC (Tomita et al. 2009). In addition, PsbQ is not required for Cl^−^ retention under Cl^−^ sufficient conditions (Miyao and Murata 1985). Consistent with this, PsbQ-deficient plants do not show apparent phenotypes when grown under normal light conditions (Ifuku et al. 2005, Yi et al. 2006). Therefore, it is not fully understood what the roles of PsbQ are in vivo. Flash oxygen yield analysis of thylakoid membranes from PsbQ-deficient *A. thaliana* suggested that the OEC was unstable compared to wild-type plants (Yi et al. 2006), seemingly consistent with the PsbQ functions observed in vitro: supporting the functions and binding of PsbP. These authors also showed that PsbQ is required for photoautotrophy under low light growth conditions (Yi et al. 2006). PSII from extreme halophytes was found to lack or have low levels of PsbQ (Andersson et al. 1984, Trotta et al. 2012), suggesting a possible in vivo role of PsbQ in supporting Cl^−^ retention. It should be noted, however, that the Cl^−^ concentration in the lumen is regulated by transporters/channels (Spetea et al. 2017, Li et al. 2021b), and the range of Cl^−^ concentration in the thylakoid lumen remains unclear (Raven 2020, Imaizumi et al. 2022).

#### PsbQ′

PsbQ′ (Ohta et al. 2003) is an extrinsic subunit unique to red-lineage PSII (Enami et al. 1995, 1998, 2005, Nagao et al. 2007) (Fig. 2). This subunit, with an apparent molecular mass of 20 kDa, is homologous to PsbQ found in green plant PSII, but the sequence similarity is low (Ohta et al. 2003). The structure of PsbQ′ is similar to that of PsbQ; it consists of a long flexible N-terminal loop and a four-helix bundle core with which it binds to the lumenal surface of CP43. There is limited information on the roles of PsbQ′. Release-reconstitution experiments showed that PsbQ′ supports the binding of PsbV and partially PsbU to PSII (Enami et al. 1998, Nagao et al. 2010). A PsbQ′-deficient mutant of a red alga, *Cyanidioschyzon merolae,* showed impaired PSII functions, but this may be due to partial dissociation of PsbV from PSII caused by the PsbQ′-deficiency (Zienkiewicz et al. 2018). FTIR analyses have shown that PsbQ′ is not directly involved in the conformational changes in the OEC, at least during the S_1_-to-S_2_ transition (Uno et al. 2013). Channel calculations based on PSII structures show that PsbQ′ could affect the O1 channel (Hussein et al. 2023). The effects of PsbQ′ on the acceptor side of PSII have been suggested by reconstituting red algal PsbQ′ to isolated cyanobacterial PSII (Yamada et al. 2018). While the redox potential of Q_A_ in the cyanobacterium *Thermosynechococcus vestitus* has been observed to be 30–40 mV lower than that in red algae (Shibamoto et al. 2010), binding of red algal PsbQ′ to *T. vestitus* PSII led to a positive shift of the redox potential of Q_A_ by approximately 32 mV(Yamada et al. 2018). This indicates that PsbQ′ can modulate the PSII acceptor side, implying a possible photoprotective function for PsbQ′.

#### Psb31

Psb31 is also an extrinsic subunit unique to red-lineage PSII (Nagao et al. 2007, Okumura et al. 2008) (Fig. 2). This protein was named ‘Psb31’ (Okumura et al. 2008) according to the nomenclature for PSII proteins (Kashino et al. 2002). It should be noted that a different protein, Sll1390 (Kashino et al. 2002), had also been named Psb31 in a PhD dissertation (Bennewitz 2009), but this protein is now known as Psb32 (Wegener et al. 2011). Psb31 has a four-helix bundle core similar to PsbQ and PsbQ′, but with a flexible C-terminal domain (Nagao et al. 2013). It binds to PSII at a lumenal pocket formed by D2, CP47, PsbH, and PsbE (Pi et al. 2019, Feng et al. 2023, You et al. 2023). The functions and properties of Psb31 have mostly been studied in the diatom *Chaetoceros gracilis*. Release-reconstitution experiments in *C. gracilis* PSII have revealed that Psb31 has unique functions in supporting the oxygen-evolving activity of PSII and in supporting the binding of PsbV and PsbU to PSII (Nagao et al. 2010). The C-terminal domain of Psb31 extends towards the OEC and contacts the Y_Z_ hydrogen-bond network (Pi et al. 2019).

The Psb31 protein was initially found in PSII from *C. gracilis* (Nagao et al. 2007, Okumura et al. 2008), and homologous genes were found in several other red-lineage algae (Okumura et al. 2008, Nagao et al. 2017). While a homologous gene was also found in a red alga *C. merolae* (Okumura et al. 2008), the presence of this subunit was not biochemically confirmed in red algae. Therefore, Psb31 was recognized as an extrinsic subunit unique to diatoms, and possibly some other red-lineage algae. However, recently, an *in situ* PSII structure from the red alga *P. purpureum* revealed that Psb31 is also present as an extrinsic subunit in red algal PSII (You et al. 2023). Here, we further investigated by BLASTp searches whether Psb31 sequences can also be found in other red algae. We found Psb31 (or at least Psb31-like) sequences in various red algae, including *Cyanidium caldarium*, *Rhodosorus marinus*, and *Galdieria sulphuraria*, in addition to the previously reported *C. merolae* and *P. purpureum*, as well as in various red-lineage algae (Supplementary Fig. S3). However, not all red algae and red-lineage algae seemed to have Psb31. For example, we failed to find Psb31 sequences in cryptophytes, consistent with a previous report which suggested that cryptophytes have lost Psb31 (Zhang et al. 2024a). It is plausible that in both red algae and red-lineage algae, some species possess Psb31, whereas others have lost it or did not acquire it. Red algae are divided into two groups, Cyanidiophyceae and Rhodophynita. Considering that *C. merolae*, *C. caldarium*, and *G. sulphuraria* belong to Cyanidiophyceae, whereas *P. purpureum* and *R. marinus* belong to Rhodophynita, Psb31 was most likely acquired by the ancestral red alga before the divergence of these two groups. It has been suggested that secondary endosymbiosis of a red alga led to the emergence of the ancestral cryptophyte, and various red-lineage algae have diverged from the ancestral cryptophytes (Penot et al. 2022). Thus, we propose that Psb31 was originally acquired by ancestral red algae and subsequently inherited by ancestral cryptophytes, which evolved from red algae through secondary endosymbiosis. Psb31 was further inherited by various red-lineage algae; however, it appears to have been lost in some red algae, cryptophytes, and some other red-lineage algae (Fig. 2b). Amino acid sequence alignments (conducted using MAFFT v7.511 [Katoh et al. 2019] after removing transit peptides predicted using TargetP-2.0 [Almagro Armenteros et al. 2019]) show that Psb31 in red algae and red-lineage algae have a well-conserved N-terminal region (Supplementary Fig. S3), which may be involved in the binding to PSII. This region, however, was not conserved in Psb31-like sequences from Dinophyceae (Okumura et al. 2008). Structural prediction by AlphaFold2 (Jumper et al. 2021) using ColabFold (Mirdita et al. 2022) suggests that Psb31 in various red algae and red-lineage algae have a similar structure with a four-helix bundle core and a flexible C-terminal domain (Fig. 3f–h). You et al. (2023) showed that the helices of the four-helix bundle core of Psb31 from the red alga *P. purpureum* were slightly longer than those from *C. gracilis* due to extension on the side with which it does not bind to PSII. Our AlphaFold2 structures show that this difference in length between red algal and red-lineage algal Psb31 is commonly observed (Fig. 3f–h). This extension of the helices increases the molecular weight of red algal Psb31 (Supplementary Table S1) (computed using Expasy [Gasteiger et al. 2005]) and is likely to affect the SDS-PAGE band patterns of PSII extrinsic subunits in red algae and red-lineage algae. Meanwhile, the computed isoelectric points (pI) show that both red algal and red-lineage algal Psb31 are basic proteins (except for *G. sulphuraria* Psb31 lacking the C-teminal domain) (Supplementary Table S1). Considering that *C. gracilis* Psb31 associates with PSII via its positively charged amino acids (Nagao et al. 2017), it can be speculated that, to some extent, red algal and red-lineage algal Psb31 have similar binding properties. Although the above data suggests the presence of Psb31 in various red algae, currently, experimental confirmation of Psb31 as a red algal PSII extrinsic subunit has only been achieved in the PSII structure from *P. purpureum* (You et al. 2023), and previous studies on the PSII extrinsic subunits in *C. caldarium* have not identified Psb31. Psb31 may be easily released from PSII during the isolation process, in line with several diatom PSII structures lacking Psb31, or Psb31 may not or only conditionally be bound to PSII in *C. caldarium*. Further studies are required to clarify the extrinsic subunit composition in red algae.

#### PsbTn

PsbTn, which used to be called the 5 kDa protein, is a small extrinsic subunit that has been found only in PSII from land plants (Ljungberg et al. 1986, Ikeuchi et al. 1989, Kapazoglou et al. 1995) (Fig. 2). It is a nuclear-encoded extrinsic subunit, different from the membrane-intrinsic subunit PsbTc; PsbTc, often referred to simply as PsbT, is widely conserved among various oxyphototrophs and is chloroplast-encoded in eukaryotes. PsbTn binds to the lumenal side of PSII in between the lumenal domains of CP47 and PsbE. This binding site overlaps with the binding site of Psb31 in red-lineage PSII. There is limited information on the functions or properties of PsbTn. Knockout of PsbTn in *A. thaliana* had little effect on plant growth (Chen et al. 2019). Although PsbTn-deficiency partially decreased the Fv/Fm and oxygen-evolving activity of PSII, it seemed to have little direct effect on electron transfer within PSII. Instead, the lack of PsbTn led to increased susceptibility of PSII to photoinhibition, and PsbTn may have a role related to photoacclimation.

PsbTn sequences and/or proteins have been reported in various angiosperms and at least one gymnosperm *Picea abies* (Opatíková et al. 2023). We conducted further investigation by BLASTp searches and found PsbTn sequences in various angiosperms, a few gymnosperms, ferns, mosses, and a multicellular streptophyte green alga (Supplementary Fig. S4). The two Cys residues in PsbTn, which form a disulfide bond, are well-conserved. While PsbTn is not present in green algae, cryo-EM structures of green algal PSII from *C. reinhardtii* and *Dunaliella salina* have suggested the presence of a protein at the same position as PsbTn in land plant PSII (Sheng et al. 2019, Caspy et al. 2023). However, the observed peptide has been annotated as a small part of *Chlamydomonas* Psb27 (also called Psb2), an assembly factor that binds to a similar position as PsbQ (Fadeeva et al. 2024). It remains to be revealed whether there really is a protein in green algal PSII that structurally replaces PsbTn.

#### CyanoP and CyanoQ

While PSII structures from thermophilic cyanobacteria have revealed the binding of three extrinsic subunits, PsbO, PsbV, and PsbU, cyanobacteria are known to also possess CyanoP and CyanoQ (Fagerlund and Eaton-Rye 2011); CyanoP is an ancestral homolog of the green plant PsbP, whereas CyanoQ is an ancestral homolog of the green plant PsbQ and red-lineage PsbQ′.

CyanoQ (Kashino et al. 2002) is a lipoprotein with lipid-modification at the N-terminal Cys residue (Thornton et al. 2004, Kashino et al. 2006), required for stable accumulation of the protein (Juneau et al. 2016). It has a four-helix bundle structure similar to the protein structures of PsbQ and PsbQ′ (Jackson et al. 2010, Michoux et al. 2014). CyanoQ has been suggested to have a role in stabilizing the binding of PsbV to PSII (Kashino et al. 2006), consistent with decreased oxygen-evolving activity by CyanoQ-deficiency, especially under low Ca^2+^ and Cl^−^ conditions (Thornton et al. 2004). It is likely that CyanoQ also has other roles in PSII, as CyanoQ has been reported to support photoautotrophy and oxygen-evolving activity in ΔPsbV mutants (Summerfield et al. 2005). Recently, binding of CyanoQ to PSII as an extrinsic subunit has been structurally confirmed in the mesophilic cyanobacteria *Synechocystis* (Gisriel et al. 2022) and *Arthrospira platensis* FACHB-439 (Zhang et al. 2024b). The binding site of CyanoQ in cyanobacterial PSII is similar to those of the four-helix bundle cores of PsbQ in green plant PSII and PsbQ′ in red-lineage PSII (Fig. 2a). Although CyanoQ has not been observed in PSII structures from the thermophilic *T. vestitus* or *T. vulcanus*, binding of CyanoQ to PSII in thermophilic cyanobacteria has been reported (Michoux et al. 2014), and it has been proposed that electrostatic interactions which drive the binding of CyanoQ to PSII are weaker in thermophilic cyanobacterial PSII (Gisriel et al. 2022).

CyanoP (De Las Rivas et al. 2004, Thornton et al. 2004) is also thought to be a lipoprotein with N-terminal lipid modification. In contrast to CyanoQ, CyanoP is likely to have a role in the assembly of PSII, rather than being an extrinsic subunit in fully active PSII (Fig. 2b). CyanoP has been found to selectively bind to inactive PSII assembly intermediates in in vitro reconstitution experiments (Cormann et al. 2014), and has been observed as a component of the PSII reaction center assembly complex, formed during the early steps of PSII assembly (Knoppová et al. 2016). This is consistent with CyanoP being closely related to PPL1 (PsbP-like protein 1), a PsbP homolog protein that serves as a PSII assembly factor in *Arabidopsis* (Ishihara et al. 2007, Che et al. 2020).

### Structural and functional complementation of the different sets of extrinsic subunits

As described above, the extrinsic subunits have crucial structural and functional roles in PSII. Therefore, although the compositions of extrinsic subunits differ among cyanobacterial, red-lineage, and green plant PSII, the different sets of extrinsic subunits should complement each other. Recently, PSII structures from various species have been revealed. Upon first inspection, the PSII structures seem to indicate that the green plant PsbO, PsbP, and PsbQ subunits simply correspond to the cyanobacterial and red-lineage PsbO, PsbV+PsbU, and CyanoQ/PsbQ′ subunits, respectively (Fig. 2a). However, more careful structural and functional comparisons of these extrinsic subunits give insight into a slightly more complex manner of complementation (Supplementary Table S2).

PsbO is commonly found in cyanobacterial, red-lineage, and green plant PSII, and although some differences are observed in its degree of contribution to the formation of a proper OEC structure (Tomita et al. 2009, Uno et al. 2013, Nagao et al. 2015), PsbOs in all oxyphototrophs have mostly similar functions.

The binding site of PsbV, found in cyanobacterial and red-lineage PSII, is occupied by PsbP in green plant PSII (Fig. 2a and Fig. 3c–e). PsbV and PsbP have similar basic functions; they both play important roles in the retention of Cl^−^ and Ca^2+^ ions in the OEC and in constructing a proper OEC structure, as suggested from FTIR (Tomita et al. 2009, Uno et al. 2013) and EPR (electron paramagnetic resonance spectroscopy) studies (Lakshmi et al. 2002).

Part of PsbU in cyanobacterial and red-lineage PSII is also replaced by PsbP in green plant PSII (Fig. 2a and Fig. 3c–e). In particular, the C-terminal region of PsbU, inserted near the Cl-2 binding site, is replaced by the Loop 4 region of PsbP (Imaizumi et al. 2022, Imaizumi and Ifuku 2022). Both the C-terminal region of PsbU (Okumura et al. 2001, 2007) and the Loop 4 region of PsbP (Imaizumi et al. 2022) have been shown to play an important role in the retention of Cl^−^ ions. PsbU in cyanobacterial and red-lineage PSII also partially overlap with the position of the N-terminal region of PsbQ in green plant PSII (Fig. 3c–e), and they may also functionally complement each other; PsbU supports the binding and ion retention functions of PsbV, whereas PsbQ supports the proper binding and ion retention functions of PsbP, likely by its N-terminal region.

CyanoQ in cyanobacterial PSII, PsbQ′ in red-lineage PSII, and PsbQ in green plant PSII have a similar four-helix bundle core that binds to the same position in PSII. While the four-helix bundle cores of these subunits complement each other structurally, it is not clear whether the association of their four-helix bundle core to CP43 has functional roles (Gisriel et al. 2022, Imaizumi and Ifuku 2022). Meanwhile, the long N-terminal loop of PsbQ′ in red-lineage PSII associates with Cyt *b*_559_ at the same position as the N-terminal region of PsbP in green-lineage PSII does (Imaizumi et al. 2025a) (Fig. 3c–e). The N-terminal regions of PsbQ′ and PsbP have similar amino acid sequences, and conserved interactions between PsbQ′/PsbP and PsbE have been observed. These conserved interactions may explain the similar transmembrane effects of these extrinsic subunits on the acceptor side of PSII (Imaizumi and Ifuku 2022, Imaizumi et al. 2025a).

## Light-Harvesting Antennas of PSII

### Variety of PSII light-harvesting antenna proteins

**Fig. 4:**
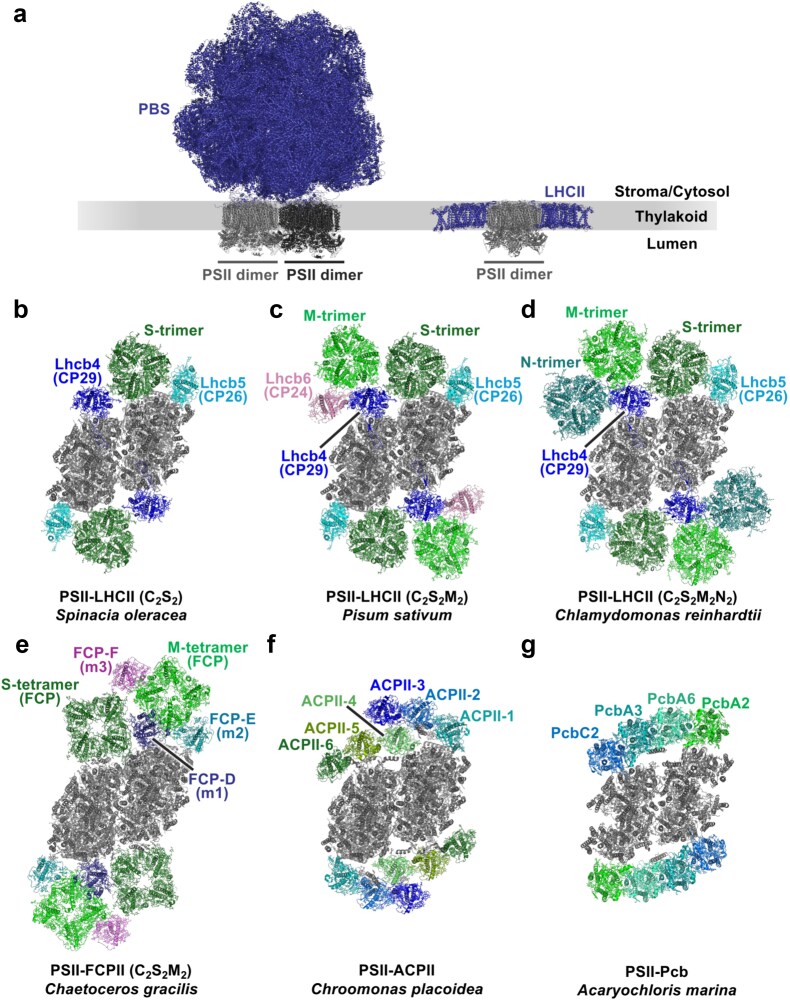
Structural diversity of PSII–light-harvesting antenna supercomplexes. (a) Comparison of PSII binding PBS and LHCII. On the left is the side view of a red algal PBS–PSII supercomplex with a hemiellipsoidal PBS (blue cartoon view) associated with two PSII dimers (gray and dark gray cartoon view) (PDB ID: 7Y5E). On the right is the side view of a land plant C_2_S_2_M_2_-type PSII–LHCII supercomplex with LHCIIs (blue cartoon view) associated with a PSII dimer (gray cartoon view) (PDB ID: 5XNL). (b–f) Comparison of the organization of LHCII proteins in various PSII–LHCII supercomplexes, viewed from the stromal side of PSII (the dimeric PSII core is shown as a gray cartoon view). (b) C_2_S_2_-type PSII–LHCII supercomplex from a land plant *Spinacia oleracea* (PDB ID: 3JCU). (c) C_2_S_2_M_2_-type PSII–LHCII supercomplex from a land plant *Pisum sativum* (PDB ID: 5XNL). (d) C_2_S_2_M_2_N_2_-type PSII–LHCII supercomplex from a green alga *Chlamydomonas reinhardtii* (PDB ID: 6KAD). (e) C_2_S_2_M_2_-type PSII–FCPII supercomplex from a diatom *Chaetoceros gracilis* (PDB ID: 7VD5). (f) PSII–ACPII supercomplex from a cryptophyte *Chroomonas placoidea* (PDB ID: 8XR6). (g) The organization of Pcb antennas in a PSII–Pcb supercomplex from a unique cyanobacteria *Acaryochloris marina* (PDB ID: 7YMI), viewed from the cytosolic side of PSII with the dimeric PSII core shown as a gray cartoon view.

To efficiently capture light energy while dissipating excess energy as heat, PSII complexes bind peripheral light-harvesting antennas. In contrast to the widely conserved PSII core, found with several different types of sets of extrinsic subunits, the light-harvesting antenna proteins, and their association with PSII are highly diverse. There are two major types of peripheral light-harvesting antennas of PSII: phycobilisomes (PBSs) (Adir et al. 2020, Bryant and Gisriel 2024) and light-harvesting complexes (LHCs) (Croce and van Amerongen 2020, Iwai et al. 2024) (Fig. 4a). PBSs are huge water-soluble pigment–protein complexes that attach to the stromal/cytosolic side of PSII. In contrast, LHCs are pigment-binding proteins embedded in the thylakoid membranes. Generally, PBSs are the major light-harvesting antennas of cyanobacterial and red algal PSII, whereas green plant and red-lineage algal PSII use LHCs (Niyogi and Truong 2013, Larkum 2020) (Fig. 2b). LHCs are also the major light-harvesting antennas of red algal, red-lineage algal, and green plant PSI. Here, we will mainly focus on the PBSs and LHCs of PSII (LHCIIs). The diversity of photoprotective mechanisms for energy dissipation has been reviewed elsewhere (Niyogi and Truong 2013, Derks et al. 2015, Goss and Lepetit 2015, Giovagnetti and Ruban 2018, Magdaong and Blankenship 2018, Larkum 2020).

PBSs are complexes of linker proteins and phycobiliproteins (PBPs), which contain bilin chromophores: phycocyanobilin (PCB), phycoviolobilin (PVB), phycoerythrobilin (PEB), and/or phycourobilin (PUB) (Bryant and Canniffe 2018). Based on their spectral properties, PBPs can be divided into four groups: phycoerythrins (PE), phycoerythrocyanins (PEC), phycocyanins (PC), and allophycocyanins (AP). The basic structural unit of PBPs is a heterodimeric (αβ) protomer, and the protomers oligomerize to toroidal (disc-shaped) trimers (αβ)_3_ or hexamers (αβ)_6_ (Bryant and Canniffe 2018, Sui 2021, Bryant and Gisriel 2024). Linker proteins can stack these discs of PBPs to form cylinders and organize the PBP discs and cylinders to form PBSs. There are several structural classes of PBSs, including hemidiscoidal, hemiellipsoidal, block-shaped, paddle-shaped, bundle-shaped, and FaRLiP (Far-Red Light Photoacclimation) bicylindrical PBSs (Bryant and Gisriel 2024). Block-shaped and hemiellipsoidal PBSs from red algae are the largest, with up to 20 MDa. In cyanobacteria, the most common PBS type is hemidiscoidal, with a size of several MDa and often composed of 2, 3, or 5 AP core cylinders and 6 to 8 peripheral rod cylinders of PC with or without PE or PEC (Bryant and Gisriel 2024). For further reading, see Adir et al. (2020), Sui (2021), and Bryant and Gisriel (2024).

LHCs are integral thylakoid membrane proteins, generally with three transmembrane α-helices that bind chlorophylls and carotenoids (Kühlbrandt et al. 1994). In green plants, LHCIIs bind Chl *a* and Chl *b*, as well as lutein, violaxanthin, and neoxanthin (Schiphorst and Bassi 2020). In contrast, LHCs in most red-lineage algae, including cryptophytes, haptophytes, and stramenopiles, bind Chl *a* and Chl *c* (Büchel 2020). In diatoms, belonging to stramenopiles, the LHCs contain fucoxanthin as the major carotenoid and are thus called fucoxanthin chlorophyll *a*/*c*-binding protein (FCP). Cryptophyte LHCs bind alloxanthin (the major carotenoid in cryptophytes) and are known as alloxanthin chlorophyll *a*/*c*-binding protein (ACP). Dinoflagellates possess the chlorophyll *a*–chlorophyll *c*_2_–peridinin protein complex (acpPC) (Hiller et al. 1993, Niedzwiedzki et al. 2014). For further reading, see Croce and van Amerongen (2020), Larkum (2020), Iwai et al. (2024).

Some species have additional or alternative unique peripheral antennas other than PBSs or LHCs. For example, prochlorophytes, a group of cyanobacteria, use membrane-intrinsic prochlorophyte chlorophyll-binding (Pcb) proteins instead of PBSs (Roche et al. 1996, Bibby et al. 2003). *Acaryochloris marina*, a chlorophyll *d*-containing cyanobacterium, also uses Pcb proteins (Chen et al. 2002, Shen et al. 2024), and only the *A. marina* MBIC11017 strain exceptionally contains PBPs (Ulrich et al. 2021). Pcb proteins are similar to CP43 and the iron-stress induced protein A (IsiA). They bind chlorophyll *a*/*b* in prochlorophytes, while they predominantly bind chlorophyll *d* in *A. marina* (Chen et al. 2005). Cryptophytes possess, in addition to ACPs, cryptophyte phycobiliproteins (Cr-PBPs), which are thylakoid lumen–localized PBPs that do not form PBSs (Gantt et al. 1971, Spear-Bernstein and Miller 1989). Dinoflagellates possess, in addition to acpPCs, peridinin-chlorophyll proteins (PCPs), which are water-soluble antenna proteins found in the thylakoid lumen (Hofmann et al. 1996). For further reading, see Larkum (2020).

### Structures of PSII–light-harvesting antenna supercomplexes in various species

High-resolution cryo-EM and cryo-ET structures of PSII associated with different peripheral light-harvesting antennas have been reported recently. The *in situ* structure of the PBS–PSII supercomplex from a cyanobacterium showed two stacked hemidiscoidal PBSs anchored to the cytosolic side of three PSII dimers forming a PSII dimer array (Zhang et al., 2024b). Each of the PBSs spanned two PSII dimers. The red alga *P. purpureum* possesses hemiellipsoidal PBSs, and cryo-ET structures of the PBS–PSII–PSI–LHC megacomplex showed PBSs tightly bound to the stromal side of PSII dimers through multiple linker proteins (You et al. 2023). These PBSs fully covered at least two PSII dimers (Fig. 4a). The structural association of PBSs to PSII dimers has been further described in detail (You et al. 2023, Bryant and Gisriel 2024, Zhang et al. 2024b).

In many land plant PSII-LHCII supercomplexes, LHCII trimers and monomers (Lhcb4 [CP29], Lhcb5 [CP26], and Lhcb6 [CP24]) bind to PSII. The antenna size of PSII depends on the light environment (Albanese et al. 2016); under low light, C_2_S_2_M_2_-type PSII–LHCII supercomplexes (Su et al. 2017) are observed, in which the dimeric PSII core (C) is surrounded by two strongly associated LHCII trimers (S) and two moderately associated LHCII trimers (M), whereas under high light, the smaller C_2_S_2_-type PSII–LHCII supercomplex (Wei et al. 2016) becomes dominant (Fig. 4b and 4c). In the green alga *C. reinhardtii*, a larger C_2_S_2_M_2_N_2_-type PSII–LHCII supercomplex has also been reported, in which naked (N), also known as loosely associated (L), LHCII trimers are bound instead of Lhcb6 (Shen et al. 2019, Sheng et al. 2019) (Fig. 4d). Interestingly, PSII-LHCII supercomplexes from a gymnosperm *P. abies* (Norway spruce), which also lack Lhcb6, were found to be able to form a similar C_2_S_2_M_2_N-type organization; however, the N-trimer was largely rotated compared to that in *Chlamydomonas* PSII–LHCII (Kouřil et al. 2020). C_2_S_2_-, C_2_S_1_M_1_-, and C_2_S_2_M_2_-type organizations have also been observed in PSII–FCPII supercomplex structures from a diatom *C. gracilis* (Nagao et al. 2019, 2022, Pi et al. 2019). However, in contrast to green plant PSII–LHCII binding LHCII trimers and monomers, *C. gracilis* PSII–FCPII bound FCPII tetramers and monomers, and the binding patterns were also different (Fig. 4e). In PSII–FCPII supercomplex structures from the diatoms *Thalassiosira pseudonana* (Feng et al. 2023) and *Cyclotella meneghiniana* (Zhao et al. 2023), FCPII was reported to bind as monomers and dimers. Structures of cryptophyte PSII–ACPII supercomplexes from *Chroomonas placoidea* (Mao et al. 2024, Zhang et al. 2024a) and *Rhodomonas salina* (Si et al. 2024) revealed that four linear trimers of ACPII attach to PSII, largely different from green plant PSII–LHCII and diatom PSII–FCPII supercomplexes (Fig. 4f). The recently characterized light-harvesting antenna of PSII in an Antarctic terrestrial green alga *Prasiola crispa* further highlights the structural diversity of LHC-type PSII antennas. In addition to typical LHCII proteins, *P. crispa* PSII employs a unique ring-like undecameric Pc-frLHC complex, composed of eleven frLHC subunits with four transmembrane helices each, to harvest far-red light (Kosugi et al. 2023, Saito et al. 2025). As for membrane-intrinsic antennas other than LHCs, PSII–Pcb structures from *A. marina* showed the binding of 8 Pcb antennas per PSII dimer; four Pcb antennas were linearly attached to one side of the PSII dimer, and symmetrically, four were attached the opposite side (Shen et al. 2024) (Fig. 4g).

## Conclusion

Intensive research, including X-ray crystallography and theoretical studies, mostly on PSII from thermophilic cyanobacteria, has led to remarkable progress in understanding the mechanisms of light-driven water oxidation. These fundamental mechanisms are most likely widely conserved among PSII from all oxyphototrophs. Meanwhile, recent advances in cryo-EM and cryo-ET have enabled the determination of PSII–light-harvesting antenna supercomplex structures from various species, giving insights into the diversity of the extrinsic subunits and light-harvesting antennas of PSII among the diverse oxygenic photosynthetic organisms. Studies on red-lineage PSII, which had been limited until recently, have especially contributed to upgrading our knowledge on the commonality and diversity of PSII. Further studies combining structural, biochemical, biophysical, and theoretical analyses on PSII from various species are expected to improve our comprehensive understanding of this ‘engine of life’ (Barber 2003). Such studies may also provide novel insights into designing efficient and stable artificial catalysts for solar-driven water splitting, with potential applications in sustainable energy production (Barber 2009, Whang and Apaydin 2018).

## Supplementary Material

pcp-2025-e-00100-File006_pcaf072

## Data Availability

The data underlying this article are available in the article and its online supplementary data.
