# Supplementary material for: Photosystem II: commonality and diversity with emphasis on the extrinsic subunits
Source: Plant Cell Physiol. 2025 Jul 2;66(11):1536–61. doi: 10.1093/pcp/pcaf072 (PMC12661325; doi:10.1093/pcp/pcaf072)
Supplement: pcp-2025-e-00100-File006_pcaf072 [file pcp-2025-e-00100-file006_pcaf072.pdf]

Supplementary Data

Photosystem II: commonality and diversity with emphasis on the extrinsic subunits

Ko Imaizumi, Kentaro Ifuku*

Division of Applied Life Sciences, Graduate School of Agriculture, Kyoto University, Kyoto 606-8502, Japan

***Corresponding author:** Kentaro Ifuku
Email: ifuku.kentaro.2m@kyoto-u.ac.jp

Contents

Supplementary Fig. S1–S4
Supplementary Table S1, S2

Supplementary Fig. S1 Distribution of lipids within PSII across the thylakoid membrane in various species.
Supplementary Fig. S2 Comparison of surface charge distributions of PSII extrinsic subunits across species.
Supplementary Fig. S3 Multiple sequence alignment of Psb31 from various red algae and red-lineage algae.
Supplementary Fig. S4 Multiple sequence alignment of PsbTn from various green plants.
Supplementary Table S1 Computed molecular weight (MW) and theoretical isoelectric point (pI) of Psb31 from red algae and red-lineage algae.
Supplementary Table S2 Structural complementation of the different sets of extrinsic subunits of PSII from various oxyphototrophs, and possible functional complementation.

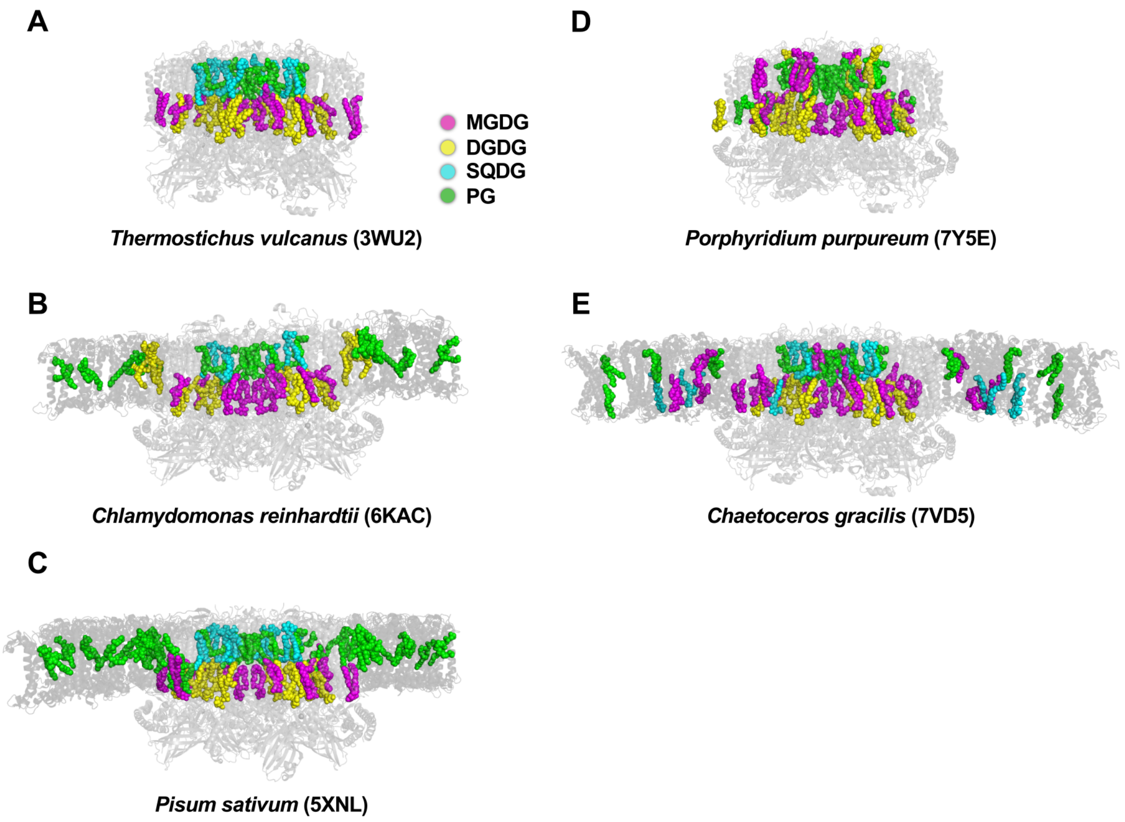

Supplementary Fig. S1 Distribution of lipids within PSII across the thylakoid membrane in various species. Side views of the structures of (A) cyanobacterial PSII, (B) green algal PSII–LHCII, (C) land plant PSII–LHCII, (D) red algal PSII, and (E) red-lineage algal PSII–FCPII with the stromal/cytosolic side upwards and lipids shown as spheres. The species and PDB ID are indicated below each PSII structure.

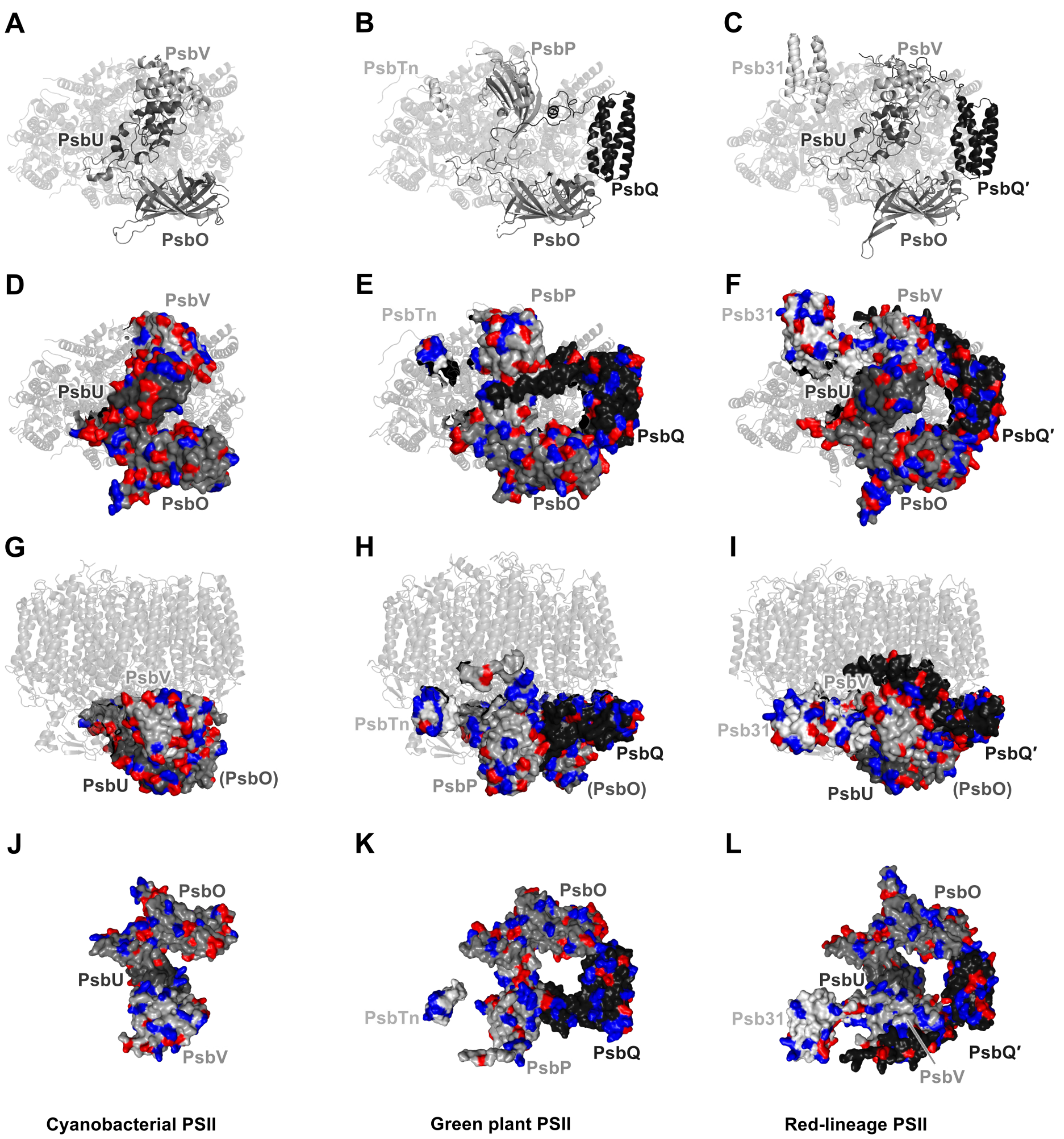

Supplementary Fig. S2 Comparison of surface charge distributions of PSII extrinsic subunits across species. (A–C) Extrinsic subunits of (A) cyanobacterial, (B) green plant, and (C) red-lineage PSII (monomers) observed from the lumenal side, shown in cartoon view. (D–L) Extrinsic subunits shown in surface representation with acidic residues colored in red, and basic residues colored in blue; (D–F) lumenal view, (G–I) side view (PsbV/PsbP at the front and PsbO at the back), and (J–L) membrane-facing view. PSII structures from (A, D, G, J) a thermophilic cyanobacterium *Thermostichus vulcanus* (PDB ID: 3WU2), (B, E, H, K) a land plant *Spinacia oleracea* (PDB ID: 8Z9D), and (C, F, I, L) a diatom *Chaetoceros gracilis* (PDB ID: 6JLU) were used. In all three species, the membrane-facing surface tends to be positively charged. Meanwhile, the charge distribution on the lumen-exposed surface seems to differ substantially among species, likely due to differences in the composition of extrinsic subunits.

```
C. merolae         DGAVSRATRERARGQYGPRVLATQAIIDRIEQAANAGNLELVAALTANPKKISKKERKELESLDGFGALYAERNAFLLLK 80
C. caldarium       DGAVSPATRQRARGVYGPRVLAYSGLIDQLSETAASGNLQRVAAMTADPSKLSKRERAALELEEGFGAFYEDKRAFSLLR 80
G. sulphuraria     DGAISFATKITSRRRYGPRILSLEQDINSLDSYIHNHEWNKVEELVSERYLNAQ-------NKPRTGKFWLERNAFQLFG 73
P. purpureum       DGAVSSATKYRARNLYGSRIFDAAPMIEKLVAAVNAKNWDTVESIV-----------------GKKGPLEDLKNSFTLFA 63
R. marinus         DGATSPATKYRVRGIYGYKIMSAGDTVNSVQGLVDSGSWVELGNLVSSA-------------KKSSGKLRDLEGALQLFK 67
C. gracilis        DGAVSSATINRARGLYGDRIAALKDAVAA----------------------------------GDFKAIAEEKNAFILFN 46
T. pseudonana      DGAKSAATQSRARGIYGQRIAALESAVNA----------------------------------GDFGAVAAEKNAFILFN 46
A. anophagefferens DGAVSAATVARSRGIYGGRIADLKDAVEK----------------------------------GDFAAVLAEKNAFDLFN 46
E. siliculosus     DGAKSLATQSRARGIYGNRIAALKGAVDK----------------------------------GDTAAIVAEQNCFRLFN 46
E. huxleyi         DGANSKATVDKARAIYGSRVVRLASASPE--------------------------------------AIIEDQNAITLFI 42
                   *** * **    *  ** ::                                                .   . .: *:

C. merolae         -GAVRG-------DRVREPEMDRVVDRLFATADEIYQAARIGNRDKVIRSARNLPLYLNSVITVAEVNPRGP-DAAPGQA 151
C. caldarium       -NAVRG-------ERTKEVEMDRVVDRFFTAVNDIHTAAEKGDADKVQRLAKDLSLYLRGVQTVADLTPSGL-ENVPGVA 151
G. sulphuraria     SGVFRD-------NKDILQRLENEEQQFFTAATRLSRAASKNDLEESERAYTDLKNAYYEFLHEAQLKTEDSVDV----- 141
P. purpureum       TGAYPT-------DKLVQTDLIGNTAVIYEMAGILRKAADQQDQKKALKAVNELVKSYKEYLAAGELKENPF-ADAPGQA 135
R. marinus         TGYFAD-------SRPKQRVMATYQSEFVDAVAALEAAAKKQDKSKANRAAAELKNAYDNIIAYAELSPNEF-ANTEGQA 139
C. gracilis        SGAYPT-------NKAKKNAAIAQTNEIFKAIRS--------------GDKAAVKSAYDAYMAANEIRPLPEINSNVGQG 105
T. pseudonana      SGAIA--------DKSKKSEAVKQTNAIFAAIRA--------------KDAGALKSAYATYKGANDIKVIVA-DKESGQG 103
A. anophagefferens SGAYAQK---SAITKAQKAIAVTETKNIMEAIAS--------------KNANQLKTSYAAYMKNAAIDTKPV-DVSTGQG 108
E. siliculosus     SGVYST-------DKAKFAEAEALAKSVVQAAGA--------------GDAKALKDSYAAYMKYTEKKSGYN-GAGDGQG 104
E. huxleyi         SGAYRAGPNGNTQDKAISKQLLALKKTALDGAKS--------------GDAAKAQGALKEMIAIAKLTEQ--------DG 100
                     .          :

C. merolae         FSSDFDWRRGRVAMHLEESAAK------GSSEMAAQPVVSKPSAPAPAPAPEVSAVGS 203
C. caldarium       SMSDYDWKRWR---KIPEIASK---------------------KLAPG--------SQ 177
G. sulphuraria     ---------------------------------------------------------- 141
P. purpureum       WSGDFSPLRKT--------------------------------GKSPY--------IE 153
R. marinus         WSSDYDYRRKF----------------------------------------------- 150
C. gracilis        YSSEFDFRSRT---KAGAIYVR------------------------------------ 124
T. pseudonana      YSNDYDFKART---TLGTIYVR------------------------------------ 122
A. anophagefferens YSNDYDWKVRT---KKGAIYQR------------------------------------ 127
E. siliculosus     FGSEFDYKNRT---PLGTVYQR------------------------------------ 123
E. huxleyi         YDGNYNPKQRR---NPGAPPTEAIVAQMGSEAYALYDRYDARSAGAPQ--------KK 147
```

Supplementary Fig. S3 Multiple sequence alignment of Psb31 from various red algae and red-lineage algae. Multiple sequence alignment was conducted using MAFFT (v7.511). The following Psb31 sequences were used. Five red algae: *Cyanidioschyzon merolae* strain 10D (XP_005537965.1), *Cyanidium caldarium* (KAK4534810.1), *Galdieria sulphuraria* (XP_005703887.1), *Porphyridium purpureum* (7Y5E_G6), and *Rhodosorus marinus* (KAJ8905369.1). Five red-lineage algae: *Chaetoceros gracilis* (BAG85209.1), *Thalassiosira pseudonana* (XP_002293679.1), *Aureococcus anophagefferens* (KAK7235713.1), *Ectocarpus siliculosus* (CBJ27783.1), and *Emiliana huxleyi* CCMP1516 (XP_005759370.1). The N-terminus of the amino acid sequences after removal of the transit peptides were decided based on prediction using TargetP-2.0 (https://services.healthtech.dtu.dk/services/TargetP-2.0/), with the support of multiple sequence alignments when necessary. Asterisks (*), colons (:), and dots (.) below the aligned sequences indicate identical, conserved, and semi-conserved amino acids, respectively. Identical amino acids are shown with black backgrounds, conserved amino acids matching the consensus sequence are shown with gray backgrounds, and conserved amino acids that do not match the consensus sequence, semi-conserved amino acids, and non-conserved amino acids matching the consensus sequence are shown with light gray backgrounds.

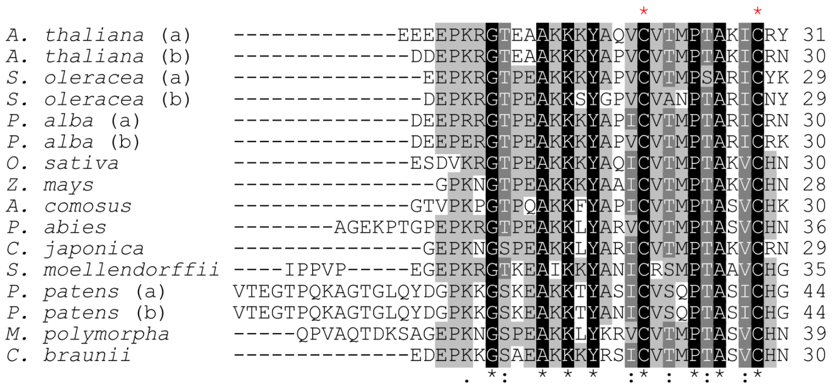

Supplementary Fig. S4 Multiple sequence alignment of PsbTn from various green plants. Transit peptide prediction and multiple sequence alignment were conducted as described in Supplementary Fig. S2. The following PsbTn sequences were used. *Arabidopsis thaliana* (a, NP_566674.2; b, NP_564589.1), *Spinacia oleracea* (a, XP_021862383.1; b, XP_021852393.1), *Populus alba* (a, XP_034896541.1; b, XP_034893560.1), *Oryza sativa* Japonica Group (XP_015623863.1), *Zea mays* (NP_001143858.1), *Ananas comosus* (XP_020085739.1), *Picea abies* (8C29_U), *Cryptomeria japonica* (XP_057845662.1), *Selaginella moellendorffii* (XP_024539502.1), *Physcomitrium patens* (a, XP_024376342.1; b, XP_024376717.1), *Marchantia polymorpha* subsp. ruderalis (OAE25500.1), and *Chara braunii* (GBG74464.1). The conserved Cys residues that form a disulfide bond are indicated with red asterisks.

Supplementary Table S1 Computed molecular weight (MW) and theoretical isoelectric point (pI) of Psb31 from red algae and red-lineage algae.

Species	MW (kDa)	Theoretical pI
C. merolae	21.8	9.47
C. caldarium	19.5	9.15
G. sulphuraria	16.6	5.46
P. purpureum	16.7	8.68
R. marinus	16.4	9.01
C. gracilis	13.3	9.52
T. pseudonana	12.8	9.58
A. anophagefferens	13.6	9.49
E. siliculosus	13.0	9.30
E. huxleyi	15.5	9.40

MW and theoretical pI were computed by Expasy's "Compute pI/Mw tool" (https://web.expasy.org/compute_pi/) using the predicted mature Psb31 sequences shown in Supplementary Fig. S2. The upper half of the table shows data on red algal Psb31 and the lower half shows data on red-lineage algal Psb31.

Supplementary Table S2 Structural complementation of the different sets of extrinsic subunits of PSII from various oxyphototrophs, and possible functional complementation.

Cyanobacterial PSII	Red-lineage PSII	Green plant PSII	Possible major complemented roles of structurally complemented regions
PsbO	PsbO	PsbO	Optimization and stabilization of the OEC
PsbV	PsbV	PsbP	Retention of Ca^{2+} and Cl^{-} ions
PsbU	PsbU	PsbQ N-terminus	Support functions and binding of PsbV/PsbP
PsbU C-terminus	PsbU C-terminus	PsbP Loop 4	Retention of Cl^{-} ions (Cl-2)
CyanoQ	PsbQ′ core	PsbQ core	Similar binding site
–	PsbQ′ N-terminus	PsbP N-terminus	Transmembrane effects on PSII acceptor side through interaction with Cyt b_{559}
–	Psb31	PsbTn (land plants)	Similar binding site
